# Challenges and Recommendations in Regulating AI Medical Devices in the European Union: A Scoping Review

**DOI:** 10.3390/healthcare14172865

**Published:** 2026-09-05

**Authors:** Guilherme Semedo, Emanuel Valpaços, Liliana Teles, Bruno Gago, Nelson Pacheco Rocha

**Affiliations:** 1 Doctorate in Business Innovation Program, University of Aveiro, 3810-193 Aveiro, Portugal; 2 Critical Catalyst, 4450-102 Matosinhos, Portugal; evalpacos@criticalcatalyst.com (E.V.); lteles@criticalcatalyst.com (L.T.); 3 Department of Medical Sciences, University of Aveiro, 3810-193 Aveiro, Portugal; bmgago@ua.pt (B.G.); npr@ua.pt (N.P.R.); 4 iBiMED—Institute of Biomedicine, University of Aveiro, 3810-193 Aveiro, Portugal; 5 IEETA—Institute of Electronics and Informatics Engineering of Aveiro, University of Aveiro, 3810-193 Aveiro, Portugal

**Keywords:** medical device, artificial intelligence, AI Act, MDR, regulation

## Abstract

**Background/Objectives**: The integration of artificial intelligence (AI) in medical devices has significant potential for transforming healthcare in the European Union (EU). However, AI medical devices are regulated under the Medical Devices Regulation (MDR) and the In Vitro Diagnostic Medical Devices Regulation (IVDR), as well as horizontal legislation such as the AI Act, resulting in a multilayered framework that raises difficulties for all stakeholders. This scoping review aims to identify and classify the challenges associated with the regulation of AI medical devices in the EU and the corresponding recommendations proposed in the academic literature. **Methods**: This review followed the PRISMA-ScR guidelines. The search was conducted in February 2026 in Web of Science, Scopus, MEDLINE PubMed, and IEEE Xplore. Challenges and recommendations were extracted through an analytical review process and grouped into secondary and primary domains, and a composite indicator was developed to assess the predominance of each domain. **Results**: Seventy studies published between 2018 and 2025 were included. A total of 261 challenges and 113 recommendations were identified and organized in five primary domains: regulatory framework, data issues, accountability and ethics, development and deployment, and certification. The regulatory framework was the domain most frequently reported, reflecting the overlap between the MDR, the AI Act, and other legislation and the absence of harmonized standards. Transparency was the only secondary domain where recommendations outnumbered challenges. **Conclusions**: This review structures the fragmented evidence into a single framework, mapping areas most frequently discussed in the literature and identifying where recommendations are still scarce, and it provides a foundation for policymaking and regulatory science on AI medical devices in the EU.

## 1. Introduction

Digital technologies and artificial intelligence (AI) are transforming medicine, medical research, and public health. The safe deployment of these technologies can help achieve the United Nations Sustainable Development Goals, including the health-related objectives of sustainable development and universal health coverage [1]. The European Union (EU) is well placed to benefit from the potential applications of AI, given its innovative research centres and startups, world-leading position in robotics, strong computing infrastructure, and large volume of public and industrial data [2]. The socioeconomic impact of AI on EU healthcare systems will be reflected in health outcomes, financial resources, and time spent by healthcare providers. MedTech Europe estimates that approximately 400,000 lives, 200 billion euros, and 1.8 billion hours of work could be saved annually [3].

AI systems can perform tasks such as visual perception, speech recognition, and translation using logic and knowledge-based approaches, statistical approaches, and machine-learning or deep learning techniques. Some AI systems demonstrate a degree of autonomy—performing tasks without constant input from a user—and adaptability—learning from experience and changing their performance [4,5]. The number of studies on the role of AI in medicine and the number of companies developing such technologies has increased substantially since the 2010s. There has been an accelerated adoption of AI in medical devices, either embedded within hardware devices or as standalone software [4].

In the EU, AI medical devices are regulated under the Medical Devices Regulation (EU) 2017/745 (MDR) and the In Vitro Diagnostic Medical Devices Regulation (EU) 2017/746 (IVDR). However, AI medical devices are additionally subjected to horizontal legislation such as the AI Act, the General Data Protection Regulation (GDPR), and the European Health Data Space (EHDS) [6,7,8,9,10].

Although legislators have worked to reduce unnecessary complexity and legal uncertainty, several stakeholders have raised concerns about the conflation of the AI Act with medical device legislation. The resulting overlap of requirements creates a complex and inconsistent landscape, delaying the delivery of safe and effective products to patients and healthcare systems [11,12,13].

Several literature reviews [14,15,16,17,18] contextualize the use of AI in healthcare, summarizing the application areas, identifying potential impacts, and suggesting future research directions. However, these studies do not consistently differentiate between general healthcare AI applications and AI medical devices, nor do they focus on regulatory or EU context. A systematic literature review [19] on trends in the use of AI in medical devices identified the main areas of application, implementation challenges, and potential strategies to accelerate the use of AI in medical devices. Similarly, a chapter in [20] identified how AI is used in medical devices and the associated limitations. Nonetheless, these studies are not focused on identifying the challenges and recommendations in regulating AI medical devices in the EU.

The literature on challenges and recommendations associated with the regulation of AI medical devices in the EU is mostly industry-based and non-academic, and a systematic academic synthesis of evidence is lacking. Therefore, this scoping review aims to identify the challenges associated with the regulation of AI medical devices in the EU and the corresponding recommendations or solutions, providing insight into the current state of the art and contributing to evidence-based decisions.

## 2. Materials and Methods

This scoping review followed the Preferred Reporting Items for Systematic reviews and Meta-Analyses extension for Scoping Reviews (PRISMA-ScR) Checklist, and a review protocol was prepared to define the (i) research questions, (ii) search strategy, (iii) inclusion and exclusion criteria, (iv) screening procedures, (v) data extraction, and (vi) synthesis and reporting [21].

This scoping review was not registered with PROSPERO, as the registry does not currently accept scoping review protocols. The review protocol was, however, developed a priori by the authors and is available upon request.

### 2.1. Research Questions

The research questions were framed using the Population, Intervention, Comparison, Outcomes, and Context (PICOC) framework, as presented in Table 1 [22].

The following research questions were elaborated:

1. RQ1: Which stakeholders are contributing to the academic research on the EU regulation of AI medical devices?
2. RQ2: What are the purposes of the current research on the regulation of AI medical devices in the EU?
3. RQ3: What are the challenges associated with the EU regulatory framework for AI medical devices?
4. RQ4: What are the proposed recommendations to address the challenges that were identified?

### 2.2. Search Strategy

The search was conducted in February 2026 in four databases, including two general databases, Web of Science and Scopus; one specific biomedical database, MEDLINE PubMed; and one specific technological database, IEEE Xplore.

Boolean queries were developed for MEDLINE PubMed using the keywords shown in Table 2 and adjusted accordingly for the other databases.

### 2.3. Inclusion and Exclusion Criteria

As inclusion criteria, the studies must address the EU regulatory framework of AI medical devices and must provide a contribution other than a simple review, including reports of challenges posed by AI medical devices.

Articles that did not address the regulation of AI medical devices, articles that did not discuss the EU regulatory context, and articles that failed to meet the specific inclusion criteria mentioned above were excluded from this review. Conference proceedings, books, book chapters, and abstracts were also excluded, as were articles not written in English and articles with no full text available.

All articles published between 1 January 2012, and 31 December 2025, were included.

### 2.4. Screening Procedures

The screening and extraction of data were performed using Microsoft Excel (v2608), and the bibliography was managed in Zotero (v7.0).

After the removal of duplicates, the articles were screened by title, excluding those clearly outside the scope of this review. Then, the abstracts were preliminarily assessed to verify whether they were related to the EU regulation of AI medical devices, excluding those that were not. The abstracts of the remaining articles were further assessed according to the inclusion and exclusion criteria. Finally, the articles that passed the abstract screening phase were thoroughly assessed against the inclusion and exclusion criteria, this time by reading the full-text version.

In all steps, the articles were independently analysed by two reviewers (G.S. and E.V.). Disagreements were discussed and resolved by consensus or, if necessary, by a third reviewer (L.T.).

### 2.5. Data Extraction

The demographic data of each study included in this review were registered in a data sheet, including the year of publication, authors, and respective affiliations. The affiliations were analysed in detail, and the country, type of organization, and area of activity of each affiliation were extracted.

The purpose and design of each study were registered. The full text of all included articles was analysed, and the challenges, obstacles, gaps, and issues related to AI medical devices were identified. The literal expressions used by the original authors were retrieved into a matrix.

Some articles presented not only challenges but also provided recommendations or solutions. Articles presenting recommendations or solutions in a systematic or structured manner were flagged.

Data extraction was performed by one reviewer (G.S.). Cases of doubt regarding the identification or classification of challenges and recommendations were discussed with two other reviewers (E.V. and L.T.) and resolved by consensus.

### 2.6. Synthesis and Reporting

Based on the demographic data of the included studies, a synthesis of their characteristics was prepared, including the distribution of studies by year and country, considering the affiliations of the respective authors. The stakeholders were characterized in terms of type of organization and area of activity based on author affiliations.

The purposes of the current research were addressed by analysing the content of the included studies in terms of both the challenges and recommendations associated with the regulation of AI medical devices.

The objective of classifying challenges and recommendations associated with the regulation of AI medical devices was to organize the fragmented evidence into a single framework. The classification was guided by an analytical review process [23]. The classification framework was developed collaboratively by the authors through iterative meetings and informal pilot testing on subsets of the included studies, with domain and subdomain definitions refined by discussions until a stable structure was reached. First, a full-text reading of the articles was conducted to identify all literal expressions used by the original authors that could be associated with challenges or recommendations. Then, each identified expression was scrutinized for its underlying theme and impact on the regulatory process. When the expressions were the same or considered very similar, they were combined into an individual challenge. When an article reported multiple distinct challenges, including those within the same subdomain, each was recorded as a separate entry. Since the research process can be viewed as a problem-solving activity [24], a similar approach was developed to identify the recommendations proposed by the included studies.

The individual challenges and recommendations were then grouped into secondary domains, which were progressively grouped into more general primary domains. The process of creating primary and secondary domains was based on the frequency of similar challenges or recommendations, their regulatory significance, and their interconnections, ensuring the encapsulation of common regulatory concerns. The development of primary and secondary domains was supported by comparative analysis with the existing literature to check for consistency and completeness and by tacit knowledge on the regulation of AI medical devices. The preliminary analysis of the literature included scientific papers found via free search, industry white papers and standards, and EU policies and guidelines. The record and grouping of individual challenges and recommendations are available in the Supplementary Materials (File S1).

To describe which primary and secondary domains are more frequently reported in the identification of challenges and recommendations, a predominance composite indicator was developed by considering the number of individual challenges or recommendations and the number of articles, following the Organization for Economic Cooperation and Development’s (OECD) handbook on constructing composite indicators [25].

First, the number of individual challenges or recommendations and the number of articles were normalized to ensure comparability. This was carried out by scaling each metric to a range of 0 to 1 using the following formula:

Normalized Value = (Value − Min Value)/(Max Value − Min Value)(1)

The values used in the composite indicator for primary domains were normalized between primary domains. The values used in the composite indicator for secondary domains were normalized between secondary domains.

Composite indicators usually rely on different weights being attributed to each metric based on their perceived importance. In the absence of a statistical or empirical basis to support weight attribution, equal weighting is considered, giving all variables the same weight. In this case, we used equal weights for the number of individual challenges or recommendations and for the number of articles.

The composite scores were calculated for each primary and secondary domain by combining the weighted normalized values:

Composite score = (W1 × Normalized number of individual challenges or recommendations) + (W2 × Normalized number of articles)(2)

W1 and W2 are equal; thus, they have been assigned a value of 0.5.

## 3. Results

### 3.1. Studies Selection

**Figure 1:**
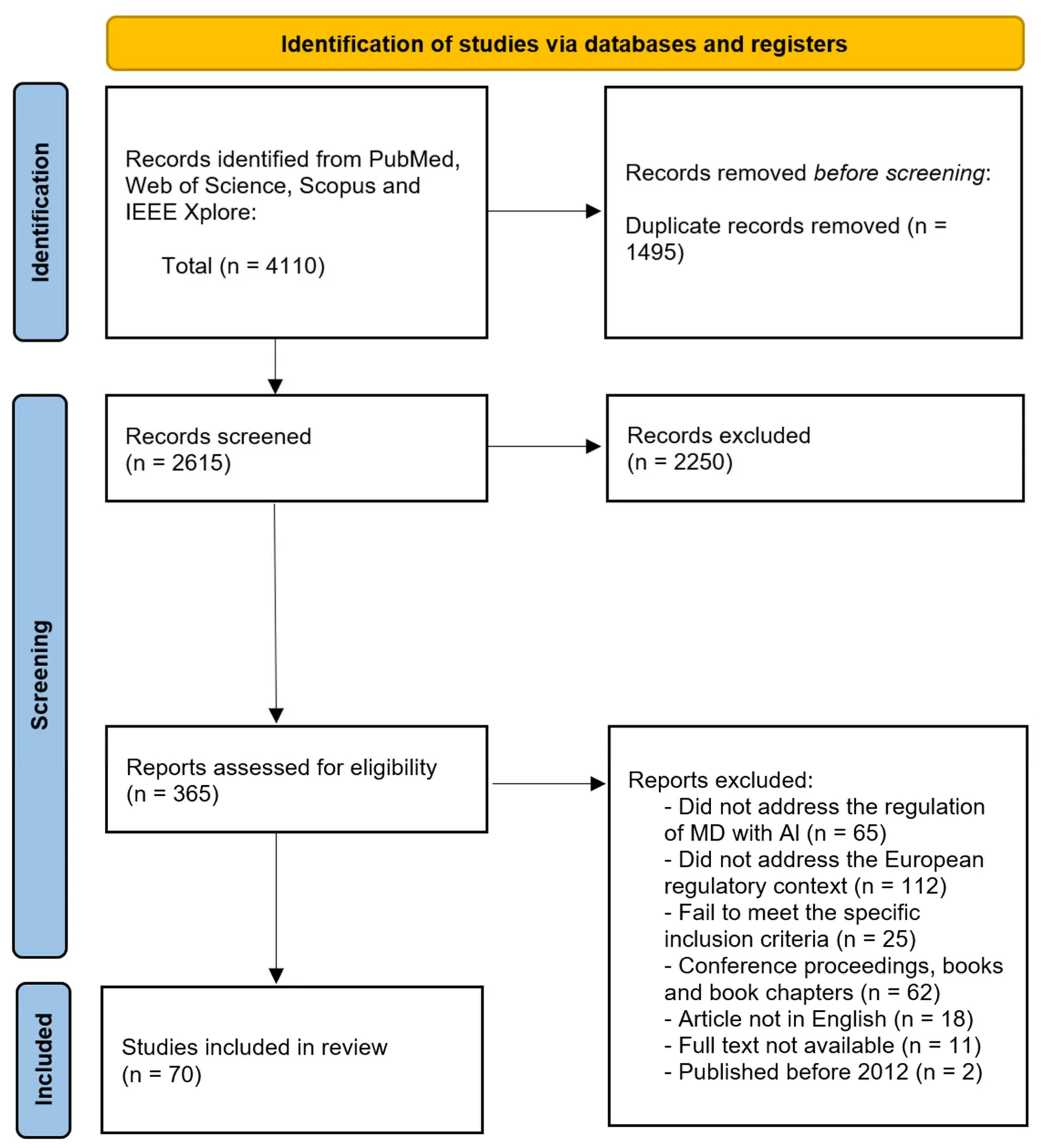
Scoping review flow diagram.

The results were reported according to PRISMA-ScR guidelines and are presented in Figure 1.

The database search was performed in February 2026, and 4110 records were retrieved. Then, 1495 records were removed because they were duplicates. In turn, during title and abstract screening, 2250 records were excluded according to the inclusion and exclusion criteria. During the full-text analysis, 295 records were excluded according to the inclusion and exclusion criteria. As a result, 70 studies were included in this scoping literature review.

### 3.2. Demographics of the Included Studies

The year of publication of the articles included in this review is presented in Table 3. The total number of articles increased from 2018 to 2025, with a small decrease in 2023.

No eligible records were published between 2012 and 2017, nor in 2019.

To analyse the origin of articles, all affiliations of lead and co-authors were considered, resulting in 391 affiliations. The results are presented in Table 4. Approximately one-third of the included articles (32.9%) have an affiliation based in Germany, comprising 22% of the total number of affiliations. Germany is followed by the UK, Italy, the Netherlands, and Belgium. Approximately two-thirds of the total number of affiliations originate from EU Member States.

### 3.3. Characterization of Stakeholders

Stakeholders were characterized through the affiliations of the authors of the included studies. The analysis reflects contributors to the academic literature rather than the full range of stakeholders affected by the regulation. The results are presented in Table 5.

Almost all articles (88.6%) included at least one affiliation from a university, comprising approximately 55% of the total number of affiliations. Academic affiliations are followed by research institutes and consulting companies.

Regarding area of activity, law and medical technological affiliations were both identified in 38.6% of the included articles, followed by technology affiliations in 31.4% and medical device affiliations in 18.6%.

Radiology was the medical specialty most frequently referenced both in terms of the number of articles and the total number of affiliations.

### 3.4. Purposes of the Research on the Regulation of AI Medical Devices in the EU

The purposes of the current research were addressed by analysing the content of the included studies in terms of challenges and recommendations associated with the regulation of AI medical devices.

After analysing the 70 articles, we identified 261 challenges and 113 recommendations, which were grouped in primary and secondary domains following the rationale presented in Table 6.

### 3.5. Challenges on the Regulation of AI Medical Devices in the EU

#### 3.5.1. Regulatory Framework

As presented in Table 7, sixty-two articles identified challenges associated with the regulatory framework, namely implementation and compliance, standards and guidelines, complexity, definitions and terminology, and information disclosure.

The regulatory framework for medical devices with AI is multilayered and fragmented. Stakeholders struggle to understand which regulations apply and how they should be applied, slowing the development of new products and significantly increasing costs [34,50,53,71,90,92].

Overlapping requirements introduce redundancies, confusion, and compliance difficulties [44,56,69,72,82,87]. Misleading overlaps are observed not only between different legislative frameworks (e.g., USA vs. EU) but also within the EU [94]. Overlap is evident between the MDR and the AI Act, for example, in their divergent approaches to risk management and post-market surveillance, as well as uncertainties on particular requirements, such as in-house exemptions [46,49,66,81,84,88,89,90]. It is also not clear how the MDR interacts with the GDPR and the EHDS [53,55,63]. These overlaps raise questions regarding how technical documentation should be combined and hinder cooperation between parties working on complex projects [44,87,90].

Time is also associated with implementation and compliance challenges. The medical devices and AI sector changes rapidly and unpredictably; thus, regulations need to be timely to be relevant. However, legislative processes are traditionally slow-paced and not adapted to AI, leaving stakeholders exposed to guidelines inconsistencies [26,28,41,61,63,67,71,83,87]. Contrasting with the time taken to regulate AI use in medical devices, deadlines established for compliance and certain conflicts of interest are criticized, making it challenging for manufacturers to maintain commercial competitiveness while remaining compliant [37,72].

Other authors raise concerns regarding disparities in digital infrastructure and political resistance among EU Member States, which impact the implementation, enforcement and monitoring of the regulation [63,71,75,80,84].

There are also specific difficulties, including the regulatory approach to software classification and challenges arising from different levels of autonomy and adaptability of the technologies [30,36,42,44]. To tackle some of these issues, the AI Act introduces regulatory sandboxes, which allow innovators to explore and experiment with new products in a controlled environment under the supervision of the regulator [96]. However, the implementation of the regulatory sandboxes is yet to be determined, and much has been left to individual member states [56,75,85].

Although the majority of authors consider that AI medical devices are heavily regulated in the EU, some suggest that certain areas might lack regulation, such as access to clinical information by AI companies and the use of synthetic data [62,64]. Others believe that the AI Act will restrict responsible operators of LLMs, while having little effect on actors operating models for irresponsible or illegal purposes [76].

Demonstrating conformity with EU Regulations relies heavily on the use of harmonized standards, which, to date, do not exist for AI medical devices [34,39]. Although the use of harmonized standards is strongly encouraged, they are not legally binding, raising questions about the extent of their effective implementation [43]. The absence of universally accepted standards for AI medical devices is particularly noticeable in the areas of risk management and cybersecurity, clinical evidence, data quality, bias, explainability, and change management [29,31,33,35,58,60,65,66,73,79,81,84,88,90]. Technical standards and guidelines addressing the responsible implementation of AI already exist; however, few are specific for medical applications [31,47]. In fact, most Medical Device Coordination Group (MDCG) guidelines do not mention AI, leading some authors to advocate for closer cooperation between the AI office and MDCG [53,60,64,72,90]. This lack of specificity creates a challenging environment for manufacturers of innovative AI solutions [39,42,44,71,79,82,92,94]. To address this, companies are using FDA guidance to work through EU regulatory processes [90].

Problems of standardization might also be related to the tasks performed by algorithms. Many of these tasks (e.g., assessments, measurements, classification) were not developed using accepted standard processes and are not maintained by standardization bodies. The conflation of tasks and algorithms can lead to issues related to responsibility, as developers are often responsible for the algorithm but not for the task [37,53].

The development of specific standards and guidelines for AI medical devices is impacted by the scarcity and unreliability of high-quality scientific literature and by the lack of a clear and agile framework for their development [30,35,39,41]. The development of standards is strongly influenced by lobbying activities, raising questions about conflicts of interest, while updating existing standards incrementally might also fail to provide the clarity and cohesiveness required for the EU market [36,47].

Due to the complexity and ambiguity of regulatory texts and overregulation, stakeholders find themselves within a labyrinth of standards, guidelines, and legal requirements that lack alignment, clarity, and technical precision [26,38,47,48,49,53,58,63,70,71,72,74,76,77,80,81,82,84,92,94,95]. There is yet scarce evidence that the EU approach to regulation will ensure the safety and performance of AI medical devices [32]. On one hand, the MDR cannot regulate AI medical devices on its own because the level of detail for software is low [36,45,61,78]. Therefore, a combination with horizontal legislation such as the AI Act is needed, which has its own complexities and technicalities [75].

The regulatory framework becomes more complex as more layers are added. For instance, for complex products, such as Advanced Therapy Medicinal Products (ATMPs) combined with AI, it is not clear how the manufacturer of the AI medical device and the Marketing Authorization Holder of the ATMP should exchange data for their respective components, who is responsible for consolidating the information (including data from clinical trials and post-market surveillance), and who provides the data to authorities [48].

The complexity and lack of detail are highlighted by the existence of devices with similar intended purposes that have undergone approval through different and more lenient pathways [32,72]. The complexity of the regulatory framework affects mainly startups and SMEs [51,95].

Words are particularly relevant in a heavily regulated environment, and this complexity is extended to definitions and terminology [70]. Missing definitions and conflating terms are common problems that arise when several domains are combined, and these inconsistencies might lead to potential loopholes [49,82]. For example, it is interesting to note that neither the MDR nor the IVDR define software, although it is mentioned approximately 50 times [33]. Ambiguities in the MDR are also evident in the rule used for the classification of software (MDR Annex VIII, Rule 11) and in the absence of any reference to AI or machine learning [30,34,36,39].

The flaws extend to the AI Act. The absence of a stable, unanimous, and international definition of AI may result in inconsistent interpretations and complicate international collaboration [26,39]. One concern is that the definition of AI in the AI Act is too broad, qualifying almost every software as an AI system and leading to overregulation [44].

The framework also lacks definitions that might impact design and development, for example, regarding bias, transparency, cybersecurity, data accuracy, synthetic data, model openness, and output interpretation [31,33,46,55,64,76].

Regarding information disclosure, both the MDR and IVDR underscore the importance of empowering patients and healthcare professionals [6,7]. Despite this, the regulations do not establish transparency of information as a fundamental principle or specify any measures to achieve it [45,59]. Most information disclosure problems are related to Eudamed, the IT system developed by the European Commission to support the implementation of the MDR and IVDR. The platform does not require manufacturers to specify that their medical devices contain AI, does not provide details regarding intended purposes, and only presents the main clinical evaluation outcomes for implantable and Class III medical devices. This not only limits the empowerment of patients and healthcare professionals but also restricts the utility of the database [53,82,83].

The AI Act also has information disclosure problems, such as those related to the poor dissemination of results gathered in regulatory sandboxes to actors other than regulators [85].

#### 3.5.2. Data Issues

Forty-five articles identified challenges associated with data issues, namely data protection and cybersecurity, data quality and completeness, dataset size and diversity, and data processing and cost (Table 8).

Challenges related to data protection and cybersecurity are particularly relevant in the healthcare sector [26,38,39,42,50,52,62,84,93].

The data used in AI models must represent the population, but governance can complicate this, particularly when data crosses international boundaries. Non-harmonized data regulations, such as GDPR and HIPAA, render universal protocols unworkable [29]. Data sharing is not only problematic when performed internationally but also locally, for example, between healthcare institutions and medical device manufacturers, as this is frequently performed using online, openly accessible tools [62]. Data protection issues are also raised when AI medical devices are made available through cloud platforms, which are not regulated as medical devices [65].

Other authors are unsure about obtaining informed consent for future data repurposing and compliance with the right to erasure [27,28,46]. This right is difficult to implement, particularly when data are already embedded into complex systems. Deleting data from backups and logs can impact conformity with legal obligations, and erasing the raw data might not fully eliminate the influence of that data on the behaviour of the model [28].

Furthermore, there are growing concerns about adversarial attacks and re-identification of patient data, especially during data export, as classical anonymization techniques are increasingly inadequate [29,34,42,45,47,49,51,63,64,68,73,80,83,92].

Implementing cybersecurity practices is therefore essential for AI medical device manufacturers, but they are also costly. Cyberthreats evolve faster than defence practices, and there is a shortage of professionals skilled in cybersecurity and AI [34,44,69]. As cybersecurity involves humans, there are concerns regarding the lack of requirements on the AI Act about organizational measures to tackle cybersecurity [55]. There was also a problem identified in the Cybersecurity Act, in which the scope of the certification framework refers directly to medical devices but not specifically to medical devices with AI [64].

Manufacturers must ensure that data used to develop AI medical devices have sufficient quality, but there are concerns of a lack of adequate data [28,33,39,49,57]. Errors in data can arise from poor sampling, cleaning, or annotation and can propagate through the system [45]. Clinical databases are typically not designed with the development of AI medical devices in mind; thus, discrepancies between clinical practice and the information captured in the database can exist. Incomplete datasets may be missing crucial variables, reducing the ability of a model to make accurate predictions [27,33,47,49]. In addition, clinical data is often siloed in different medical information systems, complicating its fast and uniform extraction [50,81].

When data is absent, there is a tendency to insert low-quality data elements such as dummy or default values, which can result in unreliable models [31].

The labelling, segmentation, and annotation of data are labour-intensive and require the expertise of medical professionals, as they are prone to human error, inconsistencies, and difficulties in tracing information [47,53,57,59]. On the other hand, the application of fully automatic data mining techniques is challenging due to the complexity of natural language, ambiguities, nuances, and medical jargon [27].

The statistical properties of data can change over time; thus, data drift and shifts might occur, making models obsolete [38,39,42,49,68,77,81,82,83,84,85,93,94]. Therefore, data quality issues ultimately extend to the users of AI medical devices. The current regulatory framework does not define specific obligations for users regarding the quality of data introduced when interacting with an AI medical device, which is most concerning for continuous or adaptive learning models [31].

The absence of well-annotated, large datasets is a major obstacle for the development of AI medical devices [26,27,43,47,61]. Data sharing resistance and lack of standardization of source data can hinder international cooperation and the gathering of sizable, quality datasets [65,80,81]. Models trained using small and poorly labelled datasets tend to overfit and have less generalizability [35,42]. Models trained on such datasets are prone to biases and may not adequately represent the target patient population, resulting in inequitable healthcare outcomes [41]. Currently, there are no specific regulations regarding the size or quality of datasets, and it is the responsibility of auditors to assess this [87].

The costs of gathering and managing a sizable dataset are high [27,28,82]. These costs include not only computational power but also the human resources involved. The preparation of data for AI models involves activities that are time-consuming and that contribute to longer project timelines [40,64,80]. There is also an insufficient incentive structure to address the issue of data processing. Manufacturers may not see the immediate financial returns of their investments, limiting the allocation of necessary resources to improve the quality of the data [31]. The cost is also a concern when manufacturers think about scaling operations [80].

#### 3.5.3. Accountability and Ethics

As presented in Table 9, forty-five articles identified challenges associated with accountability and ethics, namely transparency, representation and fairness, accountability, and ethical decision-making.

Transparency is a major concern in the current regulatory framework [26,29,30,33,35,38,39,40,41,42,44,46,47,48,49,50,52,53,59,61,62,68,69,76,81,83,84,86,87,89,93]. Language associated with transparency is inconsistent, fuzzy, and dispersed, with terms such as interpretability, explainability, causality, plausibility, traceability, comprehensibility, and understandability used interchangeably [40,42,45].

Many AI models operate as black boxes, making it difficult to understand their decision-making and raising explainability problems [27,28,31,35,40,42,47,50,62,81,83,89,93,94]. This opaqueness complicates questions related to accountability and liability and difficulties in tracing the origin of bias [33,40]. These problems are exacerbated by the lack of a defined retention period for datasets [94].

There are concerns that the regulatory framework is not equipped to deal with socio-technical and non-functional features of AI medical devices, for example, regarding representation and fairness [66]. If these aspects are not considered throughout the lifecycle of AI medical devices, systems can perpetuate social stigmas and discrimination, threatening inclusive and equitable access to healthcare services [27,33,34,45,47,50,66,83]. The outcomes of models trained on low-diversity and small datasets can be unjust, unfair, and have low applicability to sex, age, and racial and other minority subgroups [27,29,33,41,51,53,57,94]. However, ensuring that the data used for training AI models is representative of the target patient group is challenging because there is an absence of standardized criteria for determining representativeness [35].

The techniques used to process data can also have a negative impact. For example, the removal of outliers to balance datasets can inadvertently remove data from minorities or underrepresented groups. Two entities relying on the same raw data processed differently could end up with different models [31].

AI medical devices might establish correlations that are statistically significant but not clinically relevant, resulting in misleading or unfair outcomes [42]. Algorithms can also be intentionally designed in a biased manner to favour diagnoses that are more profitable or to recommend treatments produced by a particular company [27].

AI medical devices are also impacted by contextual bias, which arises when manufacturers try to transfer algorithms from one context to another, disregarding the nuances of different healthcare settings, patient populations, or medical conditions [34]. Although conformity assessments conducted by notified bodies might mitigate this, there is a general lack of understanding of the sources of bias in AI medical devices, both by the manufacturers and notified bodies [49,53].

This could happen because of the complexity of bias. Different kinds of bias can be distinguished according to their origin and to the lifecycle phase in which they are reproduced, including representation bias; measurement bias; Simpson’s paradox; sampling bias and labelling bias during data design and collection; model bias during model development; confirmation bias during model validation; and funding bias during deployment [85].

Several authors discussed issues related to accountability and liability in AI medical devices [26,27,28,29,30,31,38,42,44,45,47,52,67,68,82,83,84,86]. As AI medical devices become more autonomous, it is increasingly difficult to determine who is responsible when something goes wrong. The MDR does not contain any specific provisions on liability; thus, the general EU product liability rules apply. Apart from manufacturers, the suppliers of faulty data, notified bodies, and healthcare providers could also become liable [31]. However, the lack of transparency of AI algorithms blurs the lines of liability. Liability issues of AI were discussed in a commission report of the EU, where a risk-based revision of product liability legislation was recommended [42].

The relation between transparency and liability is particularly important when it is necessary to assess whether medical errors are attributed to healthcare providers or to the device [27,29]. This is extended to the process of obtaining informed consent regarding the treatment with an AI medical device, namely how the technology is explained and whether it is understood [28].

Accountability challenges extend to regulatory sandboxes, in which participants are exempted from administrative fines but might still face liability claims from third parties for damages [54].

Manufacturers of AI medical devices need to consider general ethical principles such as those published by the EU expert commission on AI, the WHO, and the OECD. Several countries and organizations have published similar principles, making it difficult to know which ones should be considered. In addition, these efforts are preliminary and fast-changing. The proposals converge on core values such as beneficence, respect for human autonomy and dignity, prevention of harm, justice and fairness, privacy, transparency and explicability, responsibility, and accountability. An AI medical device manufacturer that considers these values will be subjected to fewer ethical risks regarding future regulatory changes. However, measuring compliance with principles is not easy [41,72,86].

At a technical level, it is still not clear whether an AI medical device can make an ethical decision by itself. There are two ways of making ethical decisions: autonomous thinking (systematic searching for all relevant aspects of the moral problem) and heteronomous thinking (emotional, uncontrolled process reflecting what is fixed dogmatically). The output of an AI medical device does not always correspond to the most ethical choice, because it is not yet known whether morality can be translated and coded into a machine [29].

#### 3.5.4. Development and Deployment

Thirty-five articles identified challenges related to development and deployment, including clinical validation, design and development, automation and technological impact, socioeconomic and administrative, and integration and interoperability (Table 10).

Clinical validation of AI medical devices is time-consuming and financially burdensome due to difficulties in planning, data collection, testing, and high expertise required [37,47,61,90].

The MDR does not specify how to conduct studies with AI medical devices, and it must be complemented by the Clinical Trials Regulation and the AI Act. However, the responsibilities of sponsors under the regulations are not clear, and their execution raises ethical and privacy concerns [53,86].

Therefore, most AI medical devices are trained on data from a specific institution or demography, reducing their generalizability when applied to different settings or populations and raising concerns about a lack of robust clinical evidence [38,39,82]. In fact, the validation of AI medical devices is performed predominantly on independent identically distributed data and not on out-of-distribution data, meaning that models that perform well on the training dataset might not perform well on the test dataset, and those performing well on the test dataset might still not perform well in the real world [47].

Randomized clinical trials are the gold standard for establishing the performance and safety of any medical intervention, but this classical approach may not be suitable for AI, especially for continuous or adaptive learning devices that provide personalized outputs [28,43,51,90].

Clinical investigations with AI medical devices are of poor quality and limited, and their results are highly heterogeneous, complicating comparisons of trials and data aggregation for meta-analyses [43,50].

One possible approach to clinical validation could be the realization of early feasibility studies (EFSs), which are small-scale clinical investigations conducted during early development to assess initial safety and performance, especially when bench or in silico testing is insufficient. While EFSs are well established for hardware devices, their application to medical devices with AI is limited [90].

Therefore, there is a predominance of retrospective studies and studies that remain within prototyping. Valuable retrospective studies rely on data that were not specifically collected to support clinical validation. Retrospective information is also associated with more low-data-quality risks and usually requires more processing, increasing the risk of bias [43,50].

According to ISO 13485 [97], manufacturers must document design and development activities. However, the lack of technical rigor and the absence of harmonized processes within development teams can lead to delays and frictions [39]. During actual development, manufacturers face many other difficulties, such as those related to risk management, code reviews, and conflicts between rapid technological advances and the necessity to adjust test protocols [77,90].

The current approach to risk management is not tailored to AI medical devices, particularly those capable of continuous learning, because their risk profiles evolve after deployment [68]. It is difficult to frame risk management in an (already hard to determine) intended purpose; it is difficult to determine reasonably foreseeable uses, and it is difficult to identify and measure the probability and severity of harm [77,79,82,90,91,93].

The design and development of medical devices with AI are further complicated by intellectual property issues that limit access to training data and restrict collaborative innovation ecosystems. These would be partially addressed in regulatory sandboxes, but it is still unknown whether testing within a regulatory sandbox produces sufficient evidence to support conformity [67].

Manufacturers often overlook that the tools used for software development (libraries, tools for data processing, etc.) must also be validated [47]. Instead, they often rely on non-validated solutions that may not be suitable for design and development [49,77].

Artificial intelligence is having multiple impacts on society. Concerns extend from the lack of regulatory consideration of environmental aspects to the actual provision of healthcare services [63].

Medical devices with AI will impact the fiduciary relationship between clinicians and patients, reducing the confidence that patients have in healthcare providers. Interacting with AI medical devices could lessen the perceived empathy and personal care expected from healthcare providers, which can reduce patient satisfaction and impact health outcomes, given that relationship plays a role in treatment adherence and recovery [29,30,38,83].

For healthcare providers, the integration of AI in clinical workflows can lead to automation bias, deskilling, alert fatigue, and the Hawthorne effect. Less experienced healthcare providers are the most susceptible to automation bias, but this is a problem that affects clinicians of all experience levels [45,81,83,84]. In turn, healthcare providers could experience a sense of disempowerment or obsolescence, questioning their role and autonomy [29,47,50,81]. Healthcare providers may find themselves disengaged from aspects that were traditionally part of their expertise, which might deteriorate their skills over time. Deskilling has implications for patient care, especially in situations where the technology fails or is inappropriate for a specific case [42]. Healthcare professionals might also suffer from alert fatigue caused by an overwhelming number of low-priority or non-actionable alerts, and they could experience the Hawthorne effect, altering their behaviour because they are aware of being observed [83].

The regulatory framework also poses socioeconomic and administrative burdens, exposing inequalities in access to the market. The regulatory and financial burden associated with the development and deployment of AI medical devices disproportionately benefits larger companies. Although experts are primarily retained in the private sector, startups and SMEs still struggle with extensive timelines, critical infrastructure, required capital, and expertise. This bias towards larger companies stifles advances in the area, as startups and SMEs are often the ones pushing innovation [32,56,69,90]. On the other hand, smaller organizations easily scale policies and change development procedures rapidly and incrementally, while larger enterprises may struggle [94].

Additionally, navigating complex reimbursement systems is also a real barrier. In many healthcare systems, reimbursement structures are not designed to accommodate the rapid evolution of technology. Billing medical services is complex, and reimbursement systems vary between EU Member States [35,83].

In terms of integration in clinical flows, manufacturers are confronted with difficulties in fully integrating AI medical devices into already established systems, which is essential for the evaluation of workflow efficiency and software validation [27,35,89]. Healthcare providers rely on well-established protocols and guidelines for patient care, and introducing new types of data, analytics, or metrics could disrupt existing workflows [47]. Additionally, during implementation of a new solution, healthcare providers might be asked to use both systems in parallel, which is burdensome and might cause organizational resistance [27].

#### 3.5.5. Certification

As presented in Table 11, eighteen articles identified challenges associated with certification, namely organization and oversight and monitoring.

The delegation of conformity assessment to notified bodies, which are typically private entities, is a weakness of the existing regulatory framework. Notified bodies work in a competitive market and adopt market behaviours, which can conflict with their public role. Several authors point out that there is a lack of notified bodies capable of assessing the conformity of AI medical devices, resulting in unpredictable timelines [43,44,56,69,73,86,89,90].

Authors also identified challenges related to the collaboration between public entities and manufacturers, for example, regarding the value of publicly owned data and accountability issues [42,62]. Public organizations cannot compete financially with big technology companies, which concentrate experts in the private sector and create a monopoly of intellectual capacity. This extends to competent authorities, where there is a lack of specialized knowledge to enforce regulatory controls efficiently and accurately, for example, regarding the evaluation of the quality of AI training datasets [31,34,38].

There is a general lack of communication and synchronization between stakeholders, including policymakers, notified bodies, competent authorities, industry, and the public sector. Inefficiencies are evident, for example, in the creation of regulatory sandboxes, specifically in the misalignment between how the sandbox works and the actual certification process [56,69,90].

The lack of transparency in the certification process conducted by the notified bodies is a recurrent issue, creating ambiguity for manufacturers and different assessment rigor levels and delaying certifications [47,51,61,90]. Although the MDR imposed more stringent requirements regarding the appropriate expertise of notified bodies, authors remain particularly concerned about insufficient oversight of data quality and data-cleaning processes [31,36,43,49,72].

The fact that class I devices are self-certified by manufacturers without the need for an external third-party assessment raises concerns regarding the oversight of these technologies. There is a considerable risk that manufacturers, either through misinformation or with malicious intent, incorrectly classify their products [51].

### 3.6. Recommendations on the Regulation of AI Medical Devices in the EU

#### 3.6.1. Regulatory Framework

As can be seen in Table 12, seventeen articles proposed recommendations associated with the regulatory framework: implementation and compliance, standards and guidelines, complexity, definitions and terminology, and information disclosure.

The regulation of AI medical devices needs to be approached through regulatory science efforts, which can be challenging to implement because there is no central regulatory body for medical devices in the EU. This challenge could be addressed by creating specific expert panels. Currently, expert panels are defined by medical disciplines, but it would be beneficial to extend them to AI medical devices, centralizing the regulatory science expertise for all stakeholders [36,56].

Challenges related to compliance with regulations include, for example, difficulties in managing changes and implementing useful post-market surveillance (PMS) systems.

Regarding changes, the most promising approach is predetermined change control plans (PCCPs), emphasising the need to strengthen PMS activities. To gather data more efficiently, semi-automated collection systems could be implemented at the organization level rather than relying on voluntary actions by healthcare professionals and users [36,66,78,87].

Gilbert et al. recommend addressing regulatory gaps in AI medical devices not through new legislation but through specific guidance due to its greater flexibility [36,56]. The EU should not wait for international standards to be aligned. Specific guidelines on PCCPs, risk management, data, bias, and early feasibility studies are needed [58,66,89,90]. This could be achieved through the publication of MDCG guidance while ensuring that both patient representatives and experts are consulted [36,81,93,95].

Larson et al. propose extending this consensus-based process to the definition of tasks performed by an algorithm, which shall be maintained by an independent entity capable of updating them as evidence evolves [37].

Kiseleva proposed the issuance of guidelines for the development, certification, and PMS of complex combination products to ensure smooth cooperation between stakeholders responsible for different parts of a product [48].

A complex regulatory framework is prone to misalignments and gaps. To address any shortcomings, in addition to issuing guidelines, the EU could update the MDR during its next legislative review and develop common specifications for AI medical devices [56].

Achieving agreement on definitions and terms such as “cybersecurity” and “adaptive change” would facilitate a smoother transfer of information between stakeholders within a project [46,55,65].

Communication with the public is also relevant and should be transparent and informative at all levels. For example, Eudamed should disclose whether a medical device uses AI and should extend access to clinical evaluations of Class IIa and IIb devices, while manufacturers could maintain a PMS data collection platform and present the accuracy metrics of their devices to users [53,78,90].

#### 3.6.2. Data Issues

Eight articles proposed recommendations associated with data issues, including data protection and cybersecurity, data quality and completeness, dataset size, and diversity and data processing and cost (Table 13).

Best practices for classical cybersecurity include penetration testing, formal verification, and architecture design that isolates as many parts of the system as possible. These could be complemented by implementing medical machine-learning operations (MedMLOps) infrastructure, facilitating automated patient anonymization/de-identification [47,80].

Although classical cybersecurity remains necessary for safeguarding sensitive information, risks could also be tackled through the implementation of organizational measures, starting by legally recognizing the collaboration between manufacturers and public institutions [62,89]. At project level, cybersecurity organizational measures include the definition of roles (e.g., incident response and red teaming) and the provision of training [47,55].

Two authors believe that the EHDS will provide the necessary framework for ensuring data privacy [84,85].

The quality and completeness of data could be compromised by errors during the labelling of datasets. The most obvious countermeasure is to increase investment in the collection of high-quality data. Technical measures can include data from assessments of data quality using specific indicators and health-specific toolboxes [47].

Regarding dataset size, when a sufficiently large and representative dataset is unavailable, methods such as data augmentation, synthetic resampling, simulation models, and generative-adversarial-network-based synthetic data can be useful alternatives. Using synthetic data can reduce the cost of preparing databases. However, evidence is still contradictory, and many issues remain unresolved, such as the exclusive dependency on synthetic data versus the use of mixed datasets [47,64].

In addition, using learning methods such as federated and distributed learning can also alleviate the challenges associated with gathering large-scale, centralized databases. These methods allow different stakeholders to train a model together while keeping data confidential [47].

Lazcoz and de Miguel believe that limited representativeness within databases could be addressed by waiving consent regarding access to data for research purposes. This could only be performed if the use of data is for public benefit and does not pose high risks to individuals [85].

The costs associated with data management could be reduced by implementing automated data de-identification and collection. On the other hand, hospitals and healthcare professionals should be compensated for the added labor of data annotation [80].

#### 3.6.3. Accountability and Ethics

As shown in Table 14, fourteen articles proposed recommendations associated with accountability and ethics (i.e., transparency, representation, and fairness and accountability). No recommendations were proposed for the secondary ethical decision-making domain.

Kiseleva et al. argued that the AI Act should establish a clear hierarchy between transparency-related concepts. They propose that transparency is an umbrella concept and a legal principle, while neighbouring concepts (such as interpretability, explainability, causability) are measures supporting the realization of the principle.

Transparency could be divided into external (toward patients), internal (toward healthcare providers), and insider (from AI developers toward themselves) [46]. Transparency could be improved, for example, by making more information publicly available through Eudamed and ensuring that patients receive information about the potential use of their data by manufacturers of AI medical devices [59,62].

It has been suggested that the MDR should be updated to specify the information that should be provided to users, including the types of data used to train and validate the devices, as well as their risks, side effects, algorithmic changes, and any limitations in explainability. These explanations should be provided during conformity assessment and evaluated by specialized healthcare professionals. When specifying transparency requirements, the process should be participatory and negotiated between patients, health providers, manufacturers, and regulators [46,47,59,66].

In practice, an AI medical device could have embedded capacities to provide automated explanations adapted to the needs of the audience. At the technical level, transparency can be improved using techniques such as saliency maps, locally interpretable model-agnostic explanations (LIMEs), Shapley values, and example-based explanations, although some explanations can be misleading due to their simplifying nature [46,47,81]. A more rigorous and auditable approach to explainability is to establish traceability and control over the full lifecycle of data and model development, complemented by expert human-in-the-loop oversight during the training process [87,88,94].

Petersen et al. identified several initiatives to improve transparency, including the Transparent Reporting of a Multivariable Prediction Model for Individual Prognosis or Diagnosis (TRIPOD) statement, the Datasheets for Datasets proposal, the Google Model Cards initiative, dataset nutrition labels, and AI FactSheets [47].

Additionally, to mitigate the black-box properties of LLM AI medical devices, manufacturers should prioritize flexible open-source LLMs when selecting a model [76].

Regarding representation and fairness, there are concerns about the lack of emphasis on the role of notified bodies in bias assessments. A framework for auditing datasets and algorithms is suggested, as the current EU framework addresses some technical biases but neglects contextual biases, such as digital ageism [53,84].

Algorithm fairness should be defined during model training, with drifts and anomalies detected after deployment [47,94]. To develop a solution representative of the target population, it could be useful to under-represent majority groups and over-represent minority groups in the training dataset. Dataset weighting methods, such as propensity score weighting or importance weighting, can be used in this regard [47].

When data are shared with manufacturers of AI medical devices, data owners can avoid unfair exploitation by applying fair representation techniques, in which only an intermediate representation of data with protected attributes is shared [47].

Van Kolfschooten et al. argue that manufacturers of AI medical devices should run extra tests to evaluate biases [53]. Popular fairness metrics include, for example, statistical parity and equalized odds. Many of these metrics are implemented in the open-source AI Fairness 360, Fairlearn Python packages, TensorFlow Fairness Indicators, and Google’s What-if Tool [47].

Stakeholders developing an AI medical device should have appropriate access to data and the ability to cooperate. This is particularly important for complex AI medical devices, for which the roles and responsibilities of every subject should be clearly defined and distinguished [48].

Other recommendations to improve accountability relate to regulatory sandboxes. Buocz et al. indicate that participants in AI sandboxes should be exempted from the duty that could lead to an administrative fine, rather than from the administrative fine itself. The authors also recommend extending the requirement of informed consent for real-world testing of high-risk AI systems to the sandbox framework [54].

#### 3.6.4. Development and Deployment

As shown in Table 15, ten articles proposed recommendations associated with development and deployment, including those related to the following: clinical validation, design and development, automation and technological impact, and socioeconomic and administrative. No recommendations were proposed for the secondary integration and interoperability domain.

The recommendation common to all articles presenting solutions for clinical validation of AI medical devices is to address clinical validation requirements early in the project. The FDA early feasibility study program is referenced as a model for the EU to consider [37,90,92].

Living Labs are a promising solution for addressing development challenges, as they facilitate collaboration between stakeholders and support open guideline development [76,81].

Development of AI medical devices could begin with simulations using retrospective or synthetic datasets, allowing researchers to understand the model’s behaviour without exposing patients to clinical risks. For example, the generalization capabilities of a model can be assessed by including out-of-distribution data in the test dataset (e.g., from a different setting) [47,90].

After this, in silico feasibility analyses and pilot collaborations with medical centres can help to refine product design and gather data on user feasibility [90]. Larson et al. recommend defining performance metrics beyond accuracy, such as reliability, applicability, and auditability [37].

The development must be supported by methodologies designed for iteration and accountability. Agile practices such as RegOps provide a natural foundation for AI medical device development because they balance agility with regulatory rigor. Within this framework, peer review should be conducted continuously throughout development and should be extended beyond code review to include model architecture, training data, and validation processes [77].

Smith et al. present a specific solution for the risk management of AI medical devices using LLMs by acknowledging that they can generate uses beyond what manufacturers originally intended. Rather than anchoring risk management to a fixed intended purpose, the authors advocate for the evaluation of a broader range of reasonably foreseeable uses that may emerge over the lifecycle of the model [91].

The implementation of these practices by manufacturers must be complemented by investment in computational infrastructure and expertise within medical institutions [80].

Several authors note that educating healthcare professionals and patients about the capabilities and limitations of AI medical devices is vital to mitigate challenges related to technological impact [47,76,81].

The environmental impact of the technology should also be considered. AI medical devices must be as green as other goods and services in the EU and aligned with EU net-zero emissions targets [63].

Regarding socioeconomic and administrative challenges, DiGA guidance published by the German Federal Institute for Drugs and Medical Devices was highlighted as a best practice due to its clarity and specificity in handling digital health reimbursement [90].

#### 3.6.5. Certification

Nine articles proposed recommendations associated with certification, all related to the secondary organization domain (Table 16). No recommendations were proposed for the secondary oversight and monitoring domain.

The adoption of a centralised process for governing AI medical devices could be a valuable strategy to enhance efficiency. Several models have been proposed, ranging from establishing a new medical device agency with appropriate expertise to expanding the role of already existing institutions, such as the EMA or the EU Joint Research Centre [86]. In any case, efforts made by policymakers should be grounded in regulatory science [92].

On the other hand, Larson et al. state that the involvement of third parties during the assessment of AI medical devices could be beneficial. Such entities could include clinical research organizations, research laboratories, or standardization organizations. In addition, the capacity and capabilities of notified bodies must also be expanded, such as by increasing their incentives [37,56,69].

What seems consensual is the necessity to develop approaches for maximal communication efficiency between actors and flexibility by regulatory bodies (European Commission, EU AI Office, MDCG, and courts) [71,74,75,89].

### 3.7. Quantitative Analysis of Challenges and Recommendations on the Regulation of AI Medical Devices in the EU

A total of 261 challenges and 113 recommendations were identified, and the respective distribution according to primary and secondary domains is presented in Table 17.

**Figure 2:**
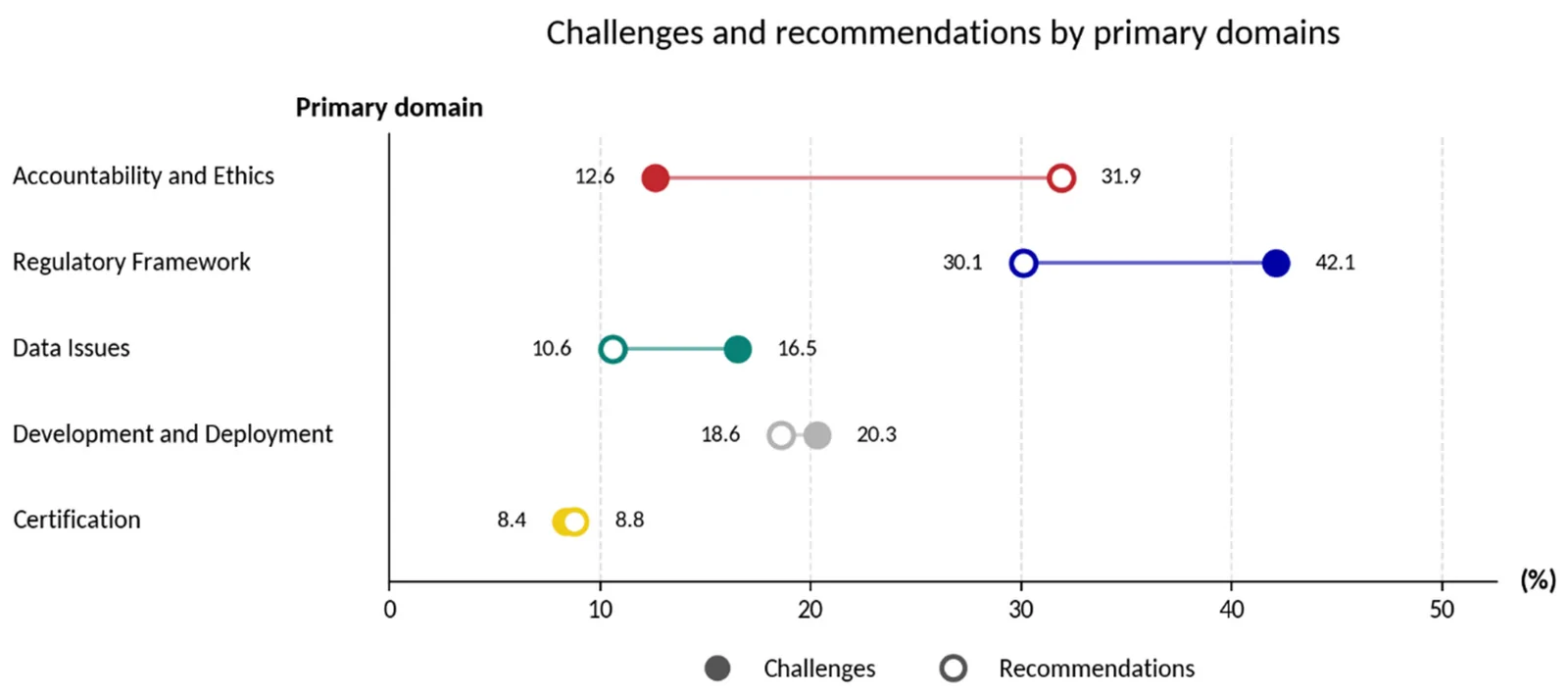
Relative percentage of challenges and recommendations for primary domains.

**Figure 3:**
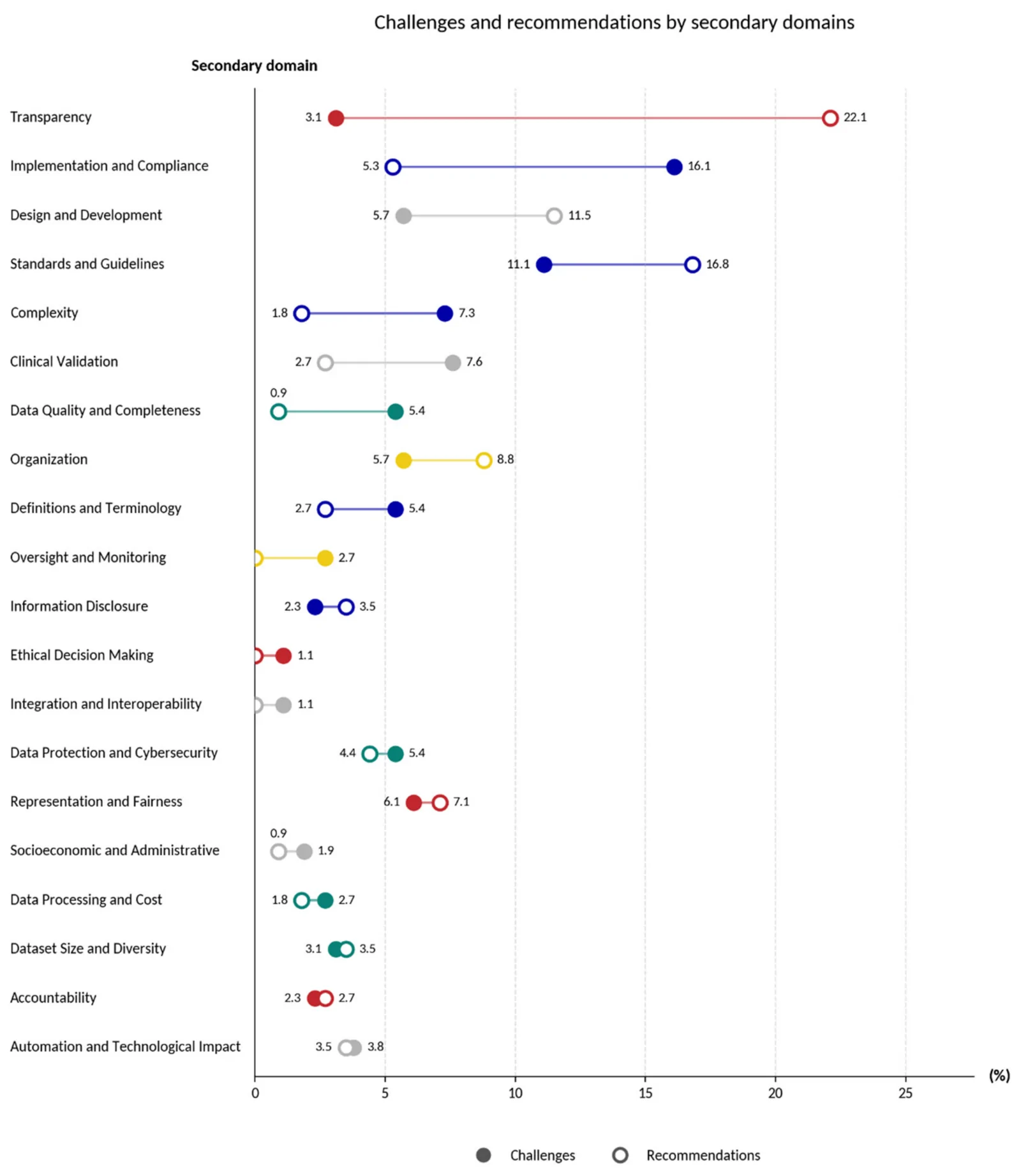
Relative percentages of challenges and recommendations for the secondary domains.

In terms of relative percentages, there are substantial differences between challenges and recommendations, as presented in Figure 2 and Figure 3, respectively. The results are organized in descending order of the absolute value of the differences between the relative percentages, which correspond to the lines between the filled and open dots.

**Figure 4:**
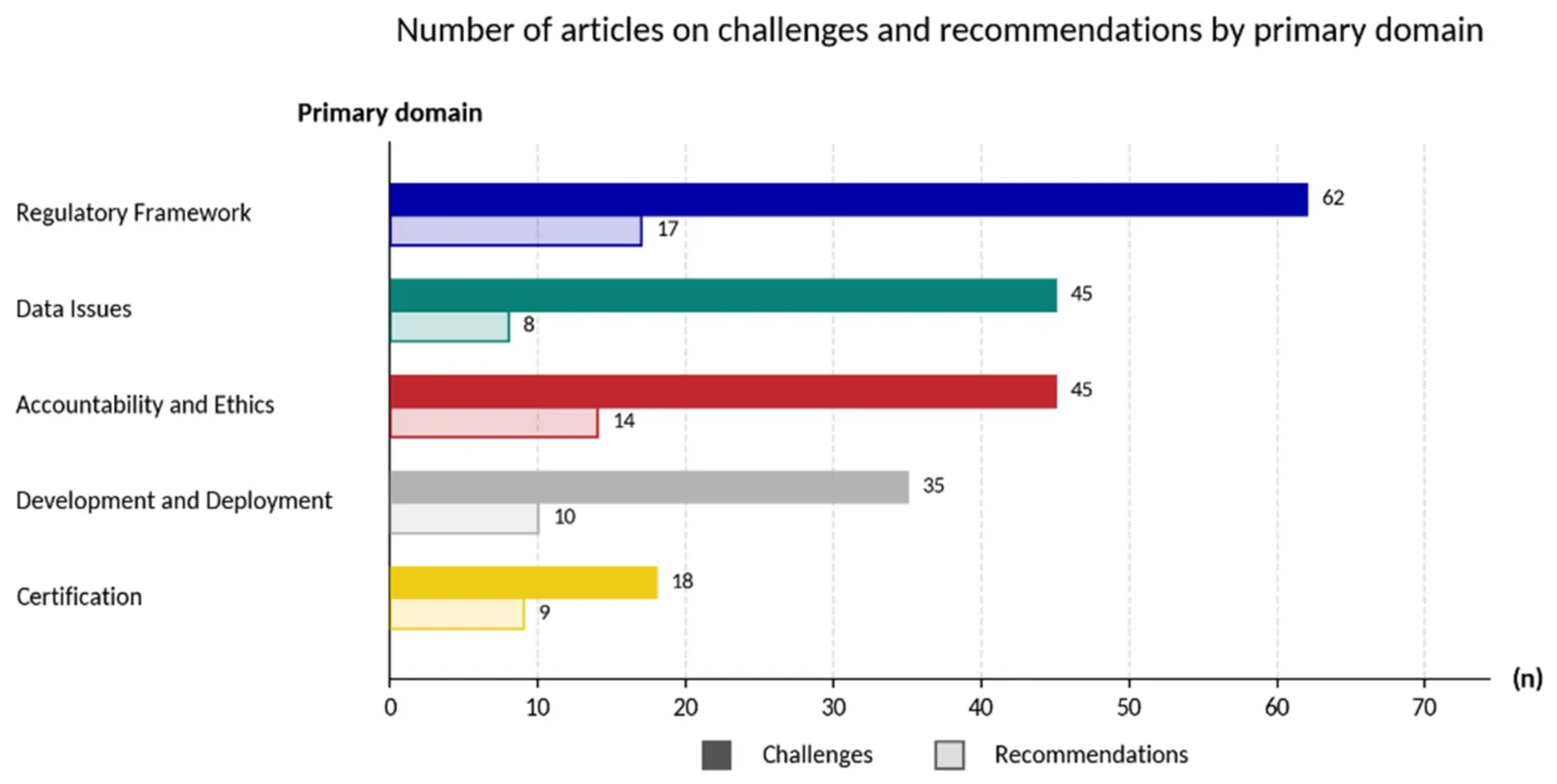
Number of articles per primary domain.

**Figure 5:**
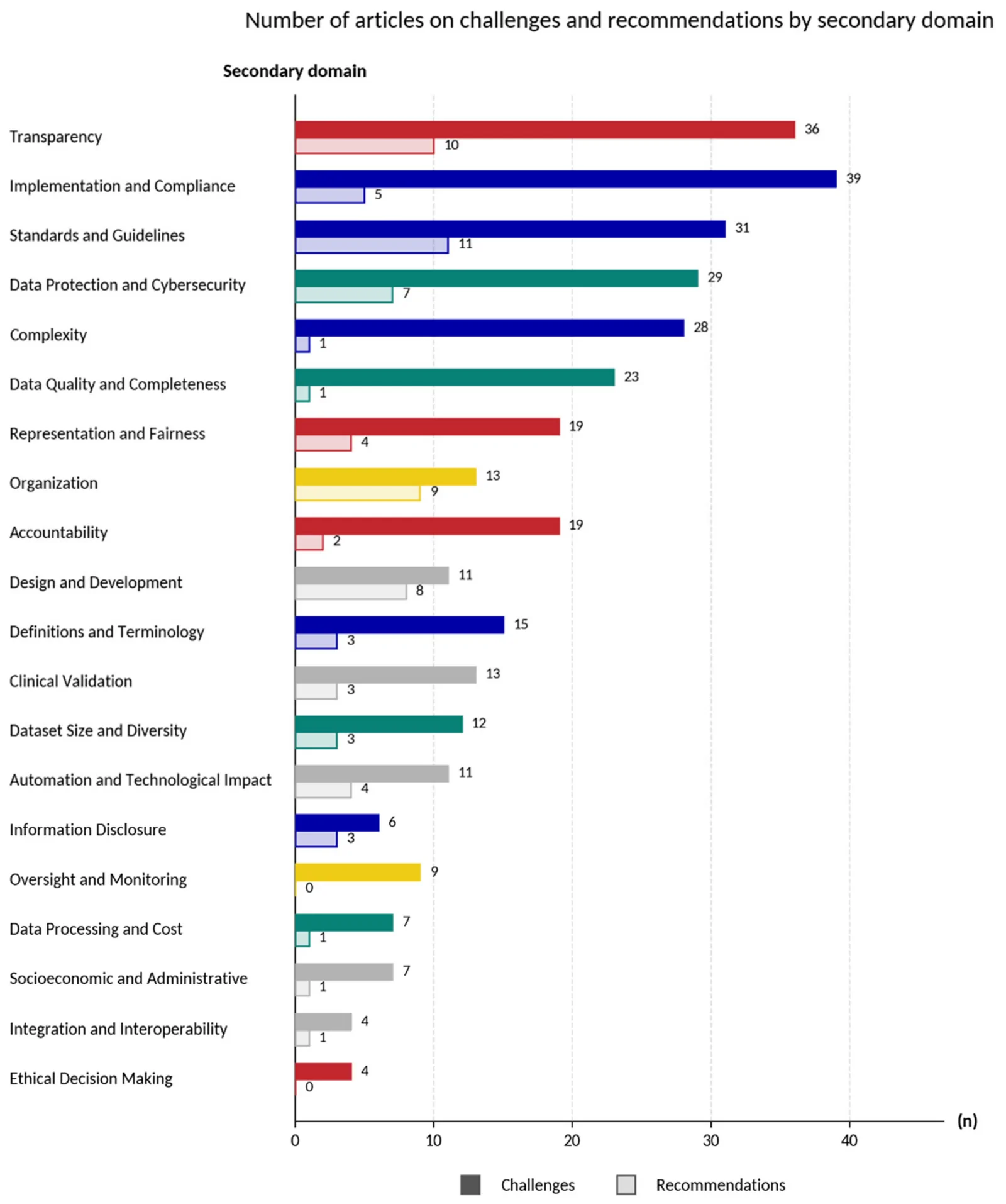
Number of articles per secondary domain.

There are also differences when comparing the number of articles referring to challenges and recommendations (Figure 4 and Figure 5). For example, 62 articles address challenges in the regulatory framework’s primary domain (i.e., 110 challenges), while 36 articles include challenges related to the secondary transparency domain (i.e., eight challenges).

**Figure 6:**
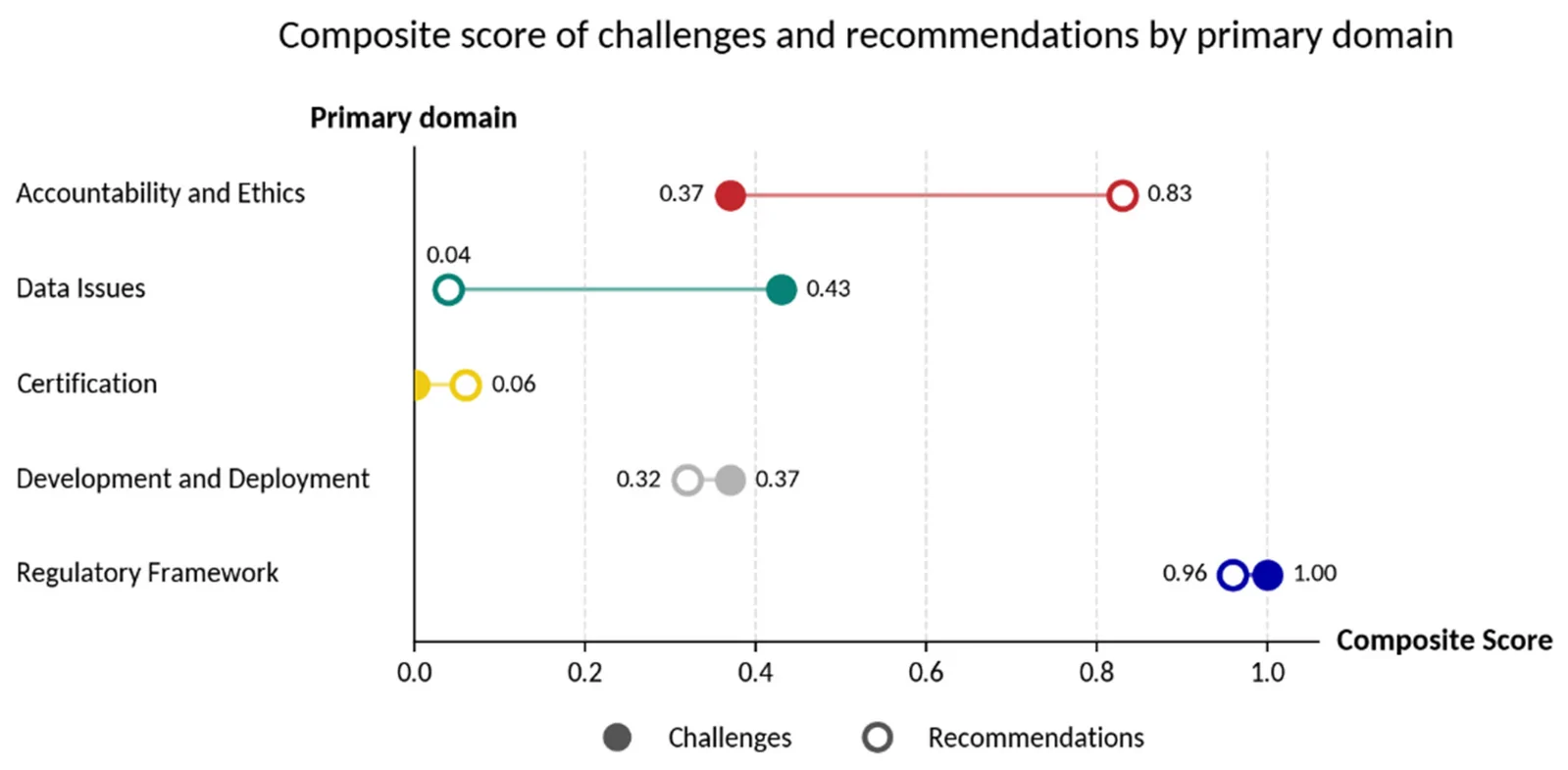
Predominance composite scores by primary domain.

**Figure 7:**
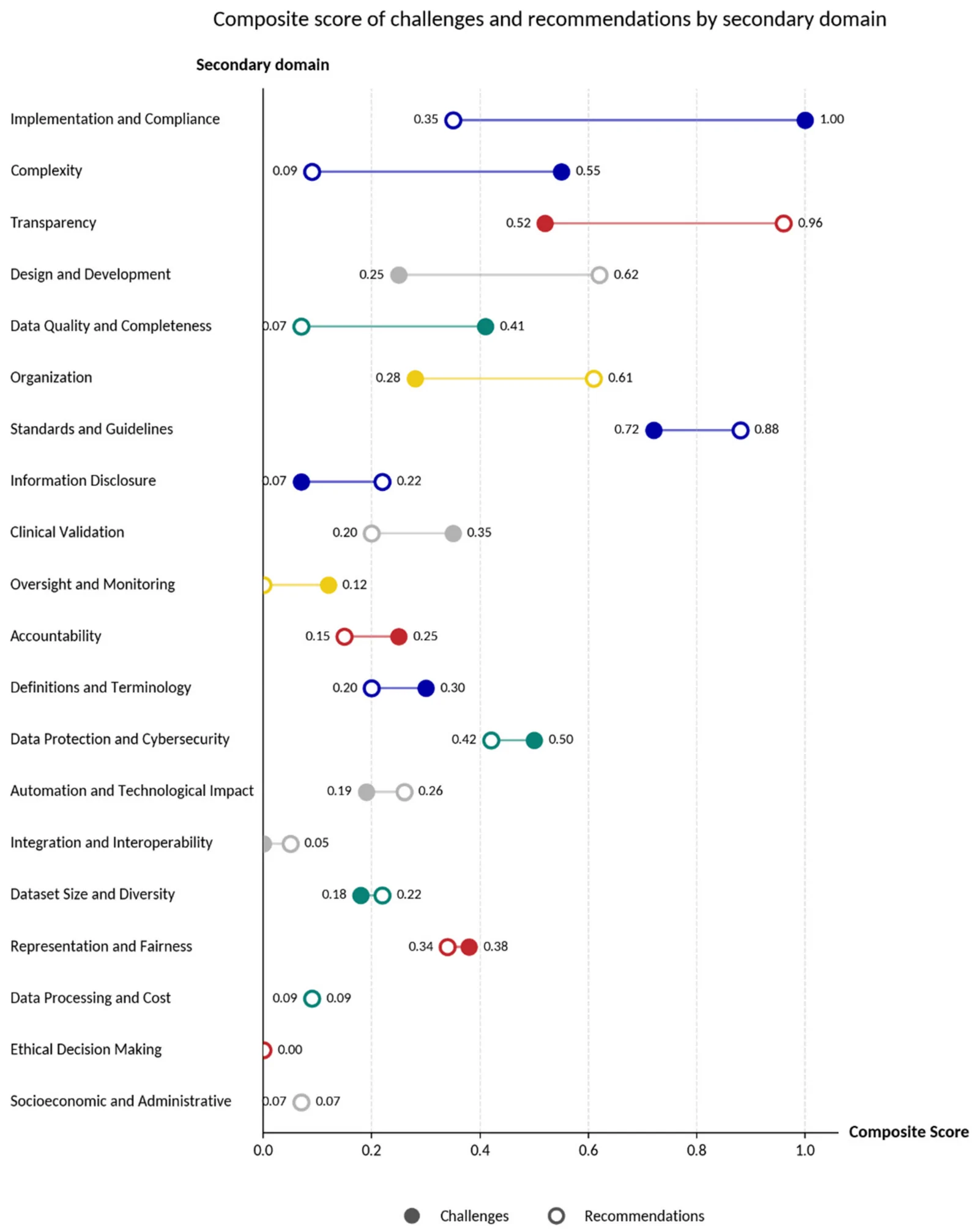
Predominance composite scores by secondary domain.

Since the number of individual challenges and recommendations identified did not always correspond to the number of articles, we found it valuable to integrate both perspectives, providing a more balanced view of how frequently each domain is discussed in the literature. To this end, we developed a composite score, as described in the Section 2. The composite score is a descriptive indicator summarizing how often challenges and recommendations were reported across the included studies. The score was constructed by the authors for the purposes of this review, and it is not a validated measure of regulatory importance, severity, or priority. The score normalizes the number of individual challenges or recommendations and the number of articles and then assigns equal weights to the normalized values. The results of the composite score for the primary and secondary domains are presented in Figure 6 and Figure 7, respectively.

The filled dots represent the composite scores for challenges, and the open dots represent the composite scores for recommendations. When the open dot lies to the left of the filled dot, the recommendations score is lower than the challenge score, indicating that the domain is predominantly discussed in terms of challenges. When the filled dot lies to the left, recommendations predominate. The distance between the two dots reflects the size of the imbalance.

## 4. Discussion

Unless otherwise indicated, statements in this section that describe the nature, magnitude, or distribution of challenges and recommendations refer to what the included studies report, whereas the interpretive comments about the structure of the evidence, its gaps, and its implications for future research are conclusions drawn by the authors of the present review. The number of articles has been increasing since 2018, with a drop in 2023. This is in line with the Artificial Intelligence Index Report of 2025, which states that publications on AI nearly doubled between 2018 and 2023. The report also shows that publications in scientific journals decreased in 2023, with authors preferring to publish in conferences and repositories [98].

Germany leads as the most prominent country mentioned in affiliations, followed by the UK, Italy, the Netherlands, and Belgium. The regulation of AI medical devices captures interest outside of the EU, namely from UK and USA researchers. The prominence of affiliations from these countries may reflect their established research infrastructure and level of expertise, a pattern that would be consistent with their market size and history of producing high-quality medical devices [99,100].

Universities were the most frequently represented type of organization mentioned in affiliations. There are also contributions from research institutes and consulting companies. The contribution from research institutes may reflect the fact that scientific publications are common outputs of research projects. The presence of consulting companies may reflect an interest in contributing expertise and maintaining visibility in this area. However, their motivation for publication cannot be determined from affiliation data alone. Given the exclusion of non-academic literature, manufacturers, notified bodies, and competent authorities might be underrepresented in this analysis, and their limited presence should not be understood as limited interest [101].

Medical IT affiliations are the most prevalent area of expertise, followed by law and IT affiliations. The strong prevalence of IT affiliations is unsurprising, as some identified issues are of technological nature. Professionals with a legal background are concerned not only with pointing out regulatory and ethical challenges but also with presenting recommendations. The involvement of professionals with diverse backgrounds suggests the relevance and potential value of interdisciplinary collaboration.

To respond to the second research question, the findings on the regulation of AI medical devices in the EU were organized within the following primary domains: regulatory framework, data issues, accountability and ethics, development and deployment, and certification. During the development of the framework, it was noted that certain categories were interconnected and that some outcomes could be included in multiple categories. This was particularly evident for aspects related to implementation and compliance, standards and guidelines, and complexity and transparency.

Regarding the third research question, there is a prevalence of challenges in the primary regulatory framework domain, which accounts for 110 of the 261 challenges identified (42.1%) and was raised by 62 of the 70 articles. This predominance is especially associated with the secondary domains of implementation and compliance, referenced by 39 articles, and standards and guidelines, referenced by 31 articles. These findings are consistent with concerns in the literature regarding fragmentation of the regulatory landscape and difficulties in interpreting regulatory requirements [102]. The regulatory burden is reported to be particularly impactful for SMEs, which may be forced to diversify their portfolios towards “non-medical” products, with a potential loss of innovative devices being placed on the market. This is particularly important considering that there are more than 35,000 medical technology companies in the EU and that SMEs make up around 92% of this number [100,103,104]. There is also a notable discrepancy between the number of articles addressing challenges versus those proposing recommendations for this domain, as 62 articles identified challenges but only 17 proposed recommendations, indicating that, within the included literature, challenges were reported more frequently than concrete recommendations. Within the included literature, there is a strong emphasis on challenges related to the representation of minorities and algorithmic fairness. This aligns with concerns raised by the European Commission and other high-level international organizations [1,105,106,107]. The debate about what constitutes ethical AI remains open, but several frameworks converge around five ethical principles: transparency, justice and fairness, non-maleficence, responsibility and privacy [108]. Considering the composite scores, it is evident that authors are also concerned with transparency challenges, since this secondary domain reaches a challenges composite score of 0.52. Within the data issues domain, the most frequently identified challenges were related to security and quality, with data protection, cybersecurity, and data quality and completeness each accounting for 14 of the 43 challenges in the data issues domain. As stated in a report commissioned by the European Parliament on the impact of the GDPR on artificial intelligence [109], data protection is at the forefront of the relationship between AI and the law, as many AI applications involve the massive processing of personal data. Data quality is also becoming a topic of focus as demands for high-quality data continue to increase, and the AI Act has been argued to be insufficient on its own to ensure data quality [110]. This issue drew concern from the European Commission, which launched a project to analyse major quality issues in 2019 and provided a set of recommendations for EU data providers [111].

Concerning the development and deployment of AI solutions, most issues relate to clinical validation, which accounts for 20 of the 53 challenges in this domain and was referenced by 13 articles, which may reflect concerns about difficulties in gathering clinical evidence and compromising the generalizability and transferability of results. The literature has identified limitations in clinical studies of AI medical devices, including concerns regarding the adequacy and completeness of the available evidence [112]. The impact of technology on the relationship between patients and healthcare providers should also not be neglected.

The challenges related to certification highlight that organizational issues appear to affect multiple stakeholder groups but are mostly associated with notified bodies. There are concerns related to the designation of Notified Bodies for AI-specific competencies, their capacity, their roles and responsibilities, and the risk of requiring separate conformity assessments and certifications [113].

Regarding the fourth research question, a total of 261 challenges were identified against 113 recommendations, a ratio of approximately 2.3 to 1 indicating an imbalance between the diagnosis of problems and the proposal of solutions. The observed 261:113 ratio between challenges and recommendations represents a mapping of the evidence reported in the included studies rather than a direct measure of the relative importance of problems and solutions, as it may also reflect differences in article length, focus, and reporting practices across the included studies.

When comparing whether the proposed recommendations align with the challenges, it can be noted that transparency received increased attention. Transparency is the secondary domain with the most recommendations, accounting for 25 of the 113 recommendations identified (22.1%) against only eight challenges, the sole secondary domain where recommendations outnumber challenges in absolute terms. It also ranks highest among secondary domains in the recommendations composite score, reaching 0.96. This possibly occurred because the articles [46,47] are very extensive, presenting multiple solutions to address transparency issues. The solutions identified in the literature appear to overlap with those recommended by EU transparency guidelines [114,115]. This concentration of solutions within transparency also explains why accountability and ethics is the only primary domain where recommendations exceed challenges, as transparency is one of its secondary domains.

The focus on recommendations regarding standards and guidelines is consistent with the challenges identified, with authors advocating for detailed guidance specific to AI medical devices. Standards and guidelines gathered 19 recommendations (16.8%), the second-highest number of any secondary domain. At the time of writing, several standards and guidelines applicable to AI medical devices are being developed or are in the process of revision, including standard IEC 62304 [116] on medical device software and ISO/IEC DTR 18988 [117] regarding the identification of processes, factors, concepts, and terms related to AI in health informatics. Greater alignment between these standards and guidelines, and with the MDR and AI Act, may help reduce inconsistencies and implementation challenges.

The solutions related to representation and fairness are also consistent with the challenges, with authors proposing concrete methods and tools to address these issues.

Despite the identification of numerous data issues, this primary domain received less emphasis regarding the solutions proposed, accounting for only 12 recommendations against 43 challenges. These data issues will be partially covered by the EHDS, which aims to provide a consistent, trustworthy, and efficient framework for the use of health data for research, innovation, policy-making, and regulatory activities. The EHDS has the potential to accelerate the development of an EU single market for digital health and data, but its successful implementation will depend on alignment with existing legislation [118,119].

The same was observed with solutions for challenges related to implementation and compliance and complexity, which accounted for six and two recommendations, respectively, with authors advocating for a regulatory science effort to determine the best approach to the EU regulation of AI medical devices.

No solutions were identified within the included literature and coding framework for challenges related to ethical decision-making, integration and interoperability, and oversight and monitoring.

When ranking the primary domains by composite score, the regulatory framework is the most predominant, both for challenges and for recommendations, with composite scores of 1.00 and 0.96, respectively. The data issues domain ranked high for challenges, with a composite score of 0.43, but it was low for recommendations, with a composite score of 0.04; moreover, in absolute terms, this domain gathered 43 challenges against only 12 recommendations, strongly favouring the identification of challenges. Accountability and ethics was the only primary domain presenting more recommendations than challenges, with 36 recommendations against 33, and it ranks high for recommendations, with a composite score of 0.83. The development and deployment domain ranks similarly for challenges and recommendations, with composite scores of 0.37 and 0.32, although challenges still predominate in absolute terms, with 53 challenges against 21 recommendations. Certification received relatively little emphasis in the included literature, ranking low both for challenges and recommendations, with a composite score of 0.06.

Ranking the secondary domains by composite score, those scoring higher for recommendations than for challenges may indicate areas in which the literature devotes relatively greater attention to potential responses. In descending order, these are as follows: transparency, design and development, organization, standards and guidelines, information disclosure, and dataset size and diversity. Excluding domains where both scores are minimal, the most balanced domains are those with minimal differences between their scores for challenges and recommendations, indicating a relative alignment between the predominance of challenges and the corresponding efforts reflected in recommendations. These are representation and fairness and automation and technological impact. Domains scoring higher for challenges than for recommendations point to areas where the reported evidence identifies more problems than proposed solutions and which may therefore warrant further attention in future research and regulatory operations. In descending order, these are as follows: implementation and compliance, complexity, data quality and completeness, clinical validation, oversight and monitoring, definitions and terminology, accountability, data protection and cybersecurity, and integration and interoperability. Among these, oversight and monitoring and integration and interoperability received no recommendations at all, placing them at the challenge end by construction rather than by reflecting a margin of effort on each side. The secondary ethical decision-making, socioeconomic and administrative, and data processing and cost domains rank low for both challenges and recommendations, indicating relatively limited attention within the included literature.

## 5. Limitations

This study has several limitations. The term AI medical device was used broadly to include various AI-based devices, such as those without adaptive learning capabilities, autonomous machine learning models, and neural networks. Similarly, the term regulatory framework was used interchangeably to refer both to the entire body of legislation applicable to AI medical devices and to specific regulatory instruments such as the MDR. Regarding the MDR, we did not distinguish between Regulation (EU) 2017/745 on medical devices and Regulation (EU) 2017/746 on in vitro diagnostic medical devices, which may overlook nuances and specific requirements unique to each regulation. Additionally, the term manufacturer was used broadly without considering that this actor is referred to as a provider in the context of the AI Act. The exclusion of non-academic sources was deliberate, as the aim was to characterize the academic evidence. However, this choice means that perspectives from the grey literature and key regulatory stakeholders, such as manufacturers, notified bodies, competent authorities, industry associations, and patient organizations, are not represented in the corpus. Since these actors are central to the practical implementation of the EU regulatory framework, the distribution and framing of challenges and recommendations reported here may differ substantially from what would emerge from a review that included their perspectives.

Given our expertise in regulatory affairs, the discussion of regulatory challenges and recommendations was likely more detailed compared to the technological aspects. We did not evaluate the methodological quality of the included studies, meaning findings from lower-quality studies could influence our conclusions. We did not apply exclusion criteria based on the number of authors per article; thus, articles with a higher number of authors may have disproportionately influenced the analysis of affiliations, skewing the prominence of contributions from certain institutions or countries. Data extraction was performed by a single reviewer, with a second opinion sought only in cases of doubt, which may have introduced extraction bias. Consequently, a formal inter-reviewer agreement analysis was not performed, and the counting of multiple challenges and recommendations within the same article is subject to the same limitation. Finally, the composite indicator developed in this review is a descriptive measure constructed by the authors for the purposes of organizing evidence, and it is not a validated measure of regulatory importance, severity, or priority. Its metrics were equally weighted due to the absence of a statistical or empirical basis for differential weighting, and its values reflect how frequently challenges and recommendations were reported in the included literature, which may be influenced by article length, focus, and reporting practices. The identification of domains as more or less prominent should therefore be read as a mapping of the reported evidence rather than as an assessment of their real-world regulatory weight.

## 6. Conclusions

This scoping review identified and classified the challenges and recommendations associated with the regulation of AI medical devices in the EU, drawing on 70 studies published between 2018 and 2025. We organized the evidence into five primary domains—regulatory framework, data issues, accountability and ethics, development and deployment, and certification—and their respective secondary domains, structuring a framework that maps a fragmented and multilayered regulatory landscape.

This review identified 261 challenges against 113 recommendations, a ratio of approximately 2.3 to 1. Within the included academic literature, challenges were identified more frequently than recommendations. The regulatory framework is the domain most frequently reported for both challenges and recommendations, with difficulties concentrated in implementation and compliance and in standards and guidelines. The overlap between the MDR, IVDR, AI Act, GDPR, and EHDS, together with the absence of harmonized standards specific to AI medical devices, emerged as the central concern across the corpus.

The distribution of recommendations does not always follow the distribution of challenges. Transparency is the only secondary domain where recommendations outnumber challenges, which also explains why the accountability and ethics domain is the only primary domain where recommendations exceed challenges. The data issues domain shows the opposite imbalance, ranking high for challenges and low for recommendations. Ethical decision-making, integration and interoperability, and oversight and monitoring received no recommendations at all, marking the areas where, within the reviewed evidence, the imbalance between reported challenges and recommendations is the largest, which may warrant further attention in future research.

Several instruments already in force or in preparation may narrow these gaps. The EHDS is expected to support the use of health data for research and regulatory activities, while the development and revision of standards such as IEC 62304, EN 18286 [120], ISO/IEC, and DTR 18988 may address the lack of specific guidance, and predetermined change control plans offer a route to manage changes in adaptive devices. Their contribution will depend on alignment with the MDR, the IVDR, and the AI Act, ensuring that the requirements are read together rather than as separate and sometimes divergent obligations.

The framework developed here offers policymakers, notified bodies, manufacturers, and researchers a structured view of where challenges and recommendations are most frequently reported in the academic literature and where solutions are still scarce. This review does not establish the effectiveness of specific regulatory interventions, nor does it prescribe the priority in which they should be addressed. Establishing effectiveness and priority would require complementary evidence from the grey literature; regulatory practice; and the direct perspectives of manufacturers, notified bodies, and competent authorities. Addressing the identified gaps in this review calls for a regulatory science effort, guidance tailored to AI medical devices, and closer cooperation between stakeholders.

## Figures and Tables

**Table 1:** PICOC framework.

	RQ1	RQ2	RQ3	RQ4
Population	Studies reporting the application of the EU’s regulatory framework to AI medical devices			
Intervention	Development and certification of AI medical devices			
Comparison	Not applicable			
Outcome	Stakeholders	Purposes of current research	Challenges	Recommendations
Context	Research papers selected from scientific databases			

Abbreviations: PICOC, Population, Intervention, Comparison, Outcomes, and Context; RQ, research question; EU, European Union; AI, artificial intelligence.

**Table 2:** Keywords for search.

Concept	Keywords
Artificial intelligence	(“Artificial Intelligence”[Mesh]) OR (“artificial intelligence”) OR (“AI”) OR (“machine-learning”)
Regulatory framework	(“regulat*”) OR (“legal*”) OR (“legislat*”) OR (“law”) OR (“rule*”) OR (“Medical Device Legislation”[Mesh]) OR (“medical device regulation”) OR (“MDR”) OR (“IVDR”) OR (“2017/745”) OR (“2017/746”)
Medical device	(“Medical Device*”) OR (“medical software”) OR (“clinical software”) OR (“medical informatic*”) OR (“MD”) OR (“MDs”) OR (“IVD”) OR (“IVDs”) OR (“app”) OR (“MDSW”) OR (“SaMD”)

Abbreviations: AI, artificial intelligence; MDR, Medical Devices Regulation (EU) 2017/745; IVDR, In Vitro Diagnostic Medical Devices Regulation (EU) 2017/746; MD, medical device; IVD, in vitro diagnostic; MDSW, medical device software; SaMD, software as a medical device; Mesh, Medical Subject Headings. The asterisk (*) is a search truncation operator retrieving all word variants sharing the preceding stem (e.g. regulat retrieves regulation, regulations, regulatory).

**Table 3:** Year of publication of the included studies.

Year of Publication	References	Number of Articles (%)
2018	[26]	1 (1.4)
2020	[27,28,29,30]	4 (5.7)
2021	[31,32,33,34,35,36,37,38,39]	9 (12.9)
2022	[40,41,42,43,44,45,46,47,48,49,50]	11 (15.7)
2023	[51,52,53,54,55,56,57]	7 (10.0)
2024	[58,59,60,61,62,63,64,65,66,67,68,69,70,71,72,73,74,75,76,77]	20 (28.6)
2025	[78,79,80,81,82,83,84,85,86,87,88,89,90,91,92,93,94,95]	18 (25.7)

**Table 4:** Countries of affiliations of the included studies.

Country of Affiliation	References	Number of Articles (%)	Number of Affiliations (%)
Germany	[35,36,42,44,45,47,50,54,55,56,57,58,59,67,70,71,75,76,78,79,81,90,93]	23 (32.9)	86 (22.0)
UK	[26,34,37,45,56,59,60,63,66,67,69,78,80,83,93]	15 (21.4)	34 (8.7)
Italy	[26,29,30,57,63,67,72,78,82,89,90,93]	12 (17.1)	31 (7.9)
Netherlands	[27,33,50,53,63,67,74,78,82,93]	10 (14.3)	28 (7.2)
Belgium	[46,48,55,64,67,73,78,86,89,93]	10 (14.3)	15 (3.8)
USA	[27,34,37,45,59,67,68,76,91]	9 (12.9)	34 (8.7)
Switzerland	[27,32,36,41,69,78,87,93]	8 (11.4)	20 (5.1)
France	[33,46,48,59,67,71,82,90]	8 (11.4)	13 (3.3)
Denmark	[34,47,67,69,91,92,93]	7 (10.0)	10 (2.6)
Ireland	[56,88,90,92,93,94]	6 (8.6)	17 (4.3)
Austria	[31,40,54,57,78]	5 (7.1)	17 (4.3)
Spain	[61,63,78,82,85]	5 (7.1)	8 (2.0)
Finland	[39,49,77,82]	4 (5.7)	13 (3.3)
Sweden	[43,67,87,89]	4 (5.7)	5 (1.3)
Greece	[78,84,95]	3 (4.3)	14 (3.6)
Australia	[27,65]	2 (2.9)	12 (3.1)
Poland	[51,57]	2 (2.9)	6 (1.5)
Portugal	[78,80]	2 (2.9)	4 (1.0)
Czechia	[52,57]	2 (2.9)	4 (1.0)
Ukraine	[28]	1 (1.4)	3 (0.8)
Norway	[89]	1 (1.4)	3 (0.8)
Japan	[38]	1 (1.4)	2 (0.5)
Malta	[67]	1 (1.4)	2 (0.5)
Turkey	[82]	1 (1.4)	2 (0.5)
Canada	[40]	1 (1.4)	1 (0.3)
Romania	[78]	1 (1.4)	1 (0.3)
Latvia	[78]	1 (1.4)	1 (0.3)
India	[62]	1 (1.4)	1 (0.3)
Slovenia	[82]	1 (1.4)	1 (0.3)
North Macedonia	[82]	1 (1.4)	1 (0.3)
Korea	[87]	1 (1.4)	1 (0.3)
Serbia	[82]	1 (1.4)	1 (0.3)

**Table 5:** Type of organization and area of activity of stakeholders.

	References	Number of Articles (%)	Number of Affiliations (%)
Type of Organization			
University	[26,27,28,30,31,32,33,34,35,36,37,38,39,40,41,42,43,45,46,47,48,49,50,51,52,53,54,55,56,57,58,59,61,62,63,64,65,66,67,68,69,71,72,74,75,76,77,78,79,82,83,84,85,87,88,89,90,91,92,93,94,95]	62 (88.6)	218 (55.8)
Research institute	[28,29,35,47,51,53,57,59,67,68,71,73,74,80,81,82,84,85,86,89,93,94]	22 (31.4)	51 (13.0)
Consulting	[27,36,39,44,47,49,50,59,63,68,70,71,82,89]	14 (20.0)	29 (7.4)
University hospital	[26,27,70,71,76,78,82,83,84,87,90,92,93]	13 (18.6)	40 (10.2)
Hospital	[26,27,50,65,68,78,80,82,84,87,89,90]	12 (17.1)	25 (6.4)
Software provider	[27,36,50,67,76,77]	5 (7.1)	12 (3.1)
Association	[45,74,78,89,93]	5 (7.1)	6 (1.5)
Public authority	[67,80,82,93]	4 (5.7)	6 (1.5)
Independent researcher	[26,59,60,67]	4 (5.7)	4 (1.0)
Area of Activity			
Technology—Medicine ^1^	[31,35,36,37,40,47,50,56,58,59,60,62,63,67,68,70,71,74,78,79,80,82,87,89,90,92,93]	27 (38.6)	94 (24.0)
Law	[28,31,32,33,34,46,48,52,53,54,55,56,59,65,67,68,69,70,77,78,85,86,89,90,91,92,93]	27 (38.6)	54 (13.8)
Technology ^1^	[31,33,36,39,40,46,47,48,49,50,56,64,66,67,72,76,77,81,88,89,94,95]	22 (31.4)	41 (10.5)
N/S	[26,30,35,38,39,49,50,53,54,55,56,57,65,67,68,71,74,90]	18 (25.7)	50 (12.8)
Medical device ^2^	[27,36,39,47,49,50,56,58,71,75,76,90,93]	13 (18.6)	18 (4.6)
Radiology	[26,32,37,50,70,78,80,89]	8 (11.4)	33 (8.4)
Ethics	[41,43,55,61,65,66,69]	7 (10.0)	13 (3.3)
Oncology	[65,70,71,78,80,86,89]	7 (10.0)	13 (3.3)
Pharmaceutical	[27,29,44,68,90]	5 (7.1)	22 (5.6)
Cardiovascular	[42,67,78,84,93]	5 (7.1)	14 (3.6)
Neurology	[36,37,76,87]	4 (5.7)	12 (3.1)
Pathology	[35,40]	2 (2.9)	7 (1.8)
Psychiatry	[45]	1 (1.4)	5 (1.3)
Intensive care	[50]	1 (1.4)	5 (1.3)
Gastroenterology	[83]	1 (1.4)	4 (1.0)
Dentistry	[57]	1 (1.4)	4 (1.0)
Ophthalmology	[51]	1 (1.4)	2 (0.5)

^1^ The category “technology—medicine” is distinct from the broader category “technology”. This area encompasses affiliations that combine elements of IT and medicine, while the “technology” category is reserved for affiliations strictly associated with technology. ^2^ The category “medical devices” is specifically reserved for affiliations related to medical devices, covering manufacturers, consultants, or technical experts. Abbreviations: N/S, not specified; IT, information technology.

**Table 6:** Rationale for the classification of data in primary and secondary domains.

Domains	Rationale
**Regulatory Framework**	Includes challenges and recommendations related to laws, regulations, standards, and guidelines that govern the development, certification, and surveillance of AI medical devices in the EU.
Implementation and Compliance	Highlights the intrinsic characteristics of the regulatory framework that dictate how the legislation is applied in practice.
Standards and Guidelines	Addresses aspects related to standards and guidance documents and their impact on the development and validation of AI medical devices.
Complexity	Arises from the necessity to streamline and simplify the regulatory landscape of AI medical devices. This category highlights the impacts of regulatory complexity on the innovation and deployment of AI medical devices.
Definitions and Terminology	Exists because there is a core set of concepts that constitute the backbone of any regulatory framework, ensuring that all stakeholders share a common understanding of requirements.
Information Disclosure	Justified by the importance of transparency in regulatory processes, the availability of information to stakeholders, and how these factors influence informed decision-making and public health.
**Data Issues**	Includes challenges and solutions related to data used in AI medical devices.
Data Protection and Cybersecurity	Arises given the highly sensitive nature of medical data. This category includes information on privacy and cybersecurity of AI medical devices.
Data Quality and Completeness	Covers aspects related to the quality of data used in AI medical devices, such as the impact of collection and processing activities.
Dataset Size and Diversity	Exists because the volume of data is an important aspect during the training of AI medical devices.
Data Processing and Cost	Focuses on the operational and financial aspects of data management in AI medical devices.
**Accountability and Ethics**	Includes challenges and solutions related to ethical aspects of AI medical devices.
Transparency	Addresses aspects related to the “black box” nature of AI medical devices and how this complicates their explainability.
Representation and Fairness	Focuses on the implications of representation and fairness throughout the lifecycle of AI medical devices, including bias.
Accountability	Exists because as AI medical devices become more autonomous, it is important to determine accountability in cases of errors or adverse events.
Ethical Decision-Making	Explores broader ethical considerations of AI medical devices.
**Development and Deployment**	Includes challenges and recommendations related to all aspects of the development of AI medical devices. This primary domain should also include aspects influencing the deployment of AI medical devices in clinical settings.
Clinical Validation	Arises from the necessity of rigorous clinical validation of AI medical devices. This category includes aspects related to validation in clinical settings and the types of studies required for clinical validation.
Automation and Technological Impact	Explores the psychological and social consequences of AI medical devices on patient–provider dynamics or in the environment.
Integration and Interoperability	Addresses the technical aspects of incorporating AI medical devices in diverse clinical workflows and software environments.
Socioeconomic and Administrative	Highlights economic and administrative challenges associated with the development and deployment of AI medical devices.
Design and Development	Focuses on broader aspects of AI medical device verification and validation.
**Certification**	Includes challenges and recommendations related to certification processes.
Organization	Arises from the need for a well-defined structural framework to ensure that entities have the necessary independence and resources to effectively regulate and oversee AI medical devices.
Oversight and Monitoring	Addresses aspects related to the certification processes conducted by certification entities.

Abbreviations: AI, artificial intelligence; EU, European Union.

**Table 7:** Articles identifying challenges related to the regulatory framework.

Domains	References	Number of Articles (%)
Regulatory Framework	[26,28,29,30,31,32,33,34,35,36,37,38,39,41,42,43,44,45,46,47,48,49,50,51,53,54,55,56,58,59,60,61,62,63,64,65,66,67,69,70,71,72,73,74,75,76,77,78,79,80,81,82,83,84,85,87,88,89,90,92,94,95]	62 (88.6)
Implementation and Compliance	[26,28,30,34,36,37,41,42,44,46,49,50,53,54,55,56,61,62,63,64,66,67,69,71,72,75,76,80,81,82,83,84,85,87,88,89,90,92,94]	39 (55.7)
Standards and Guidelines	[29,30,31,33,34,35,36,37,39,41,42,43,44,47,53,58,60,64,65,66,71,72,73,79,81,82,84,88,90,92,94]	31 (44.3)
Complexity	[26,32,36,38,45,47,48,49,51,53,58,61,63,70,71,72,74,75,76,77,78,80,81,82,84,92,94,95]	28 (40.0)
Definitions and Terminology	[26,30,31,33,34,36,39,44,46,49,55,64,70,76,82]	15 (21.4)
Information Disclosure	[46,53,59,82,83,85]	6 (8.6)

**Table 8:** Articles identifying challenges related to data issues.

Domains	References	Number of Articles (%)
Data Issues	[26,27,28,29,31,33,34,35,38,39,40,41,42,43,44,45,46,47,49,50,51,52,53,55,57,59,61,62,63,64,65,68,69,73,77,80,81,82,83,84,85,87,92,93,94]	45 (64.3)
Data Protection and Cybersecurity	[26,27,28,29,34,38,39,42,44,45,46,47,49,50,51,52,55,62,63,64,65,68,69,73,80,83,84,92,93]	29 (41.4)
Data Quality and Completeness	[27,28,31,33,38,39,42,45,47,49,50,53,57,59,68,77,81,82,83,84,85,93,94]	23 (32.9)
Dataset Size and Diversity	[26,27,35,41,42,43,47,61,65,80,81,87]	12 (17.1)
Data Processing and Cost	[27,28,31,40,64,80,82]	7 (10.0)

**Table 9:** Articles identifying challenges related to accountability and ethics.

Domains	References	Number of Articles (%)
Accountability and Ethics	[26,27,28,29,30,31,33,34,35,38,39,40,41,42,44,45,46,47,48,49,50,51,52,53,54,57,59,61,62,66,67,68,69,72,76,81,82,83,84,85,86,87,89,93,94]	45 (64.3)
Transparency	[26,27,28,29,30,31,33,35,38,39,40,41,42,44,45,46,47,48,49,50,52,53,59,61,62,68,69,76,81,83,84,86,87,89,93,94]	36 (51.4)
Representation and Fairness	[27,29,31,33,34,35,41,42,45,47,49,50,51,53,57,66,83,85,94]	19 (27.1)
Accountability	[26,27,28,29,30,31,38,42,44,45,47,52,54,67,68,82,83,84,86]	19 (27.1)
Ethical Decision-Making	[29,41,72,86]	4 (5.7)

**Table 10:** Articles identifying challenges related to development and deployment.

Domains	References	Number of Articles (%)
**Development and Deployment**	[27,28,29,30,32,35,37,38,39,42,43,45,47,49,50,51,53,56,61,63,67,68,69,77,79,81,82,83,84,86,89,90,91,93,94]	35 (50.0)
Clinical Validation	[28,37,38,39,43,47,50,51,53,61,82,86,90]	13 (18.6)
Design and Development	[39,47,49,67,68,77,79,82,90,91,93]	11 (15.7)
Automation and Technological Impact	[29,30,38,42,45,47,50,63,81,83,84]	11 (15.7)
Socioeconomic and Administrative	[32,35,56,69,83,90,94]	7 (10.0)
Integration and Interoperability	[27,35,47,89]	4 (5.7)

**Table 11:** Articles identifying challenges related to certification.

Domains	References	Number of Articles (%)
**Certification**	[31,34,36,38,42,43,44,47,49,51,56,61,69,72,73,86,89,90]	18 (25.7)
Organization	[31,34,38,42,43,44,56,62,69,73,86,89,90]	13 (18.6)
Oversight and Monitoring	[31,36,43,47,49,51,61,72,90]	9 (12.9)

**Table 12:** Articles proposing recommendations related to the regulatory framework.

Domains	References	Number of Articles (%)
**Regulatory Framework**	[36,37,46,48,53,55,56,58,65,66,78,81,87,89,90,93,95]	17 (24.3)
Implementation and Compliance	[36,56,66,78,87]	5 (7.1)
Standards and Guidelines	[36,37,48,56,58,66,81,89,90,93,95]	11 (15.7)
Complexity	[56]	1 (1.4)
Definitions and Terminology	[46,55,65]	3 (4.3)
Information Disclosure	[53,78,90]	3 (4.3)

**Table 13:** Articles proposing recommendations related to data issues.

Domains	References	Number of Articles (%)
**Data Issues**	[47,55,62,64,80,84,85,89]	8 (11.4)
Data Protection and Cybersecurity	[47,55,62,80,84,85,89]	7 (10.0)
Data Quality and Completeness	[47]	1 (1.4)
Dataset Size and Diversity	[47,64,85]	3 (4.3)
Data Processing and Cost	[80]	1 (1.4)

**Table 14:** Articles proposing recommendations related to accountability and ethics.

Domains	References	Number of Articles (%)
**Accountability and Ethics**	[46,47,48,53,54,59,62,66,76,81,84,87,88,94]	14 (20.0)
Transparency	[46,47,59,62,66,76,81,87,88,94]	10 (14.3)
Representation and Fairness	[47,53,84,94]	4 (5.7)
Accountability	[48,54]	2 (2.9)

**Table 15:** Articles proposing recommendations related to development and deployment.

Domains	References	Number of Articles (%)
Development and Deployment	[37,47,63,76,77,80,81,90,91,92]	10 (14.3)
Clinical Validation	[37,90,92]	3 (4.3)
Design and Development	[37,47,76,77,80,81,90,91]	8 (11.4)
Automation and Technological Impact	[47,63,76,81]	4 (5.7)
Socioeconomic and Administrative	[90]	1 (1.4)

**Table 16:** Articles proposing recommendations related to certification.

Domains	References	Number of Articles (%)
**Certification**	[37,56,69,71,74,75,86,89,92]	9 (12.9)
Organization	[37,56,69,71,74,75,86,89,92]	9 (12.9)

**Table 17:** Number of individual challenges and recommendations identified per primary and secondary domain.

Domains	Number of Challenges (%)	Number of Recommendations (%)
**Regulatory Framework**	110 (42.1)	34 (30.1)
Implementation and Compliance	42 (16.1)	6 (5.3)
Standards and Guidelines	29 (11.1)	19 (16.8)
Complexity	19 (7.3)	2 (1.8)
Definitions and Terminology	14 (5.4)	3 (2.7)
Information Disclosure	6 (2.3)	4 (3.5)
**Data Issues**	43 (16.5)	12 (10.6)
Data Protection and Cybersecurity	14 (5.4)	5 (4.4)
Data Quality and Completeness	14 (5.4)	1 (0.9)
Dataset Size and Diversity	8 (3.1)	4 (3.5)
Data Processing and Cost	7 (2.7)	2 (1.8)
**Accountability and Ethics**	33 (12.6)	36 (31.9)
Transparency	8 (3.1)	25 (22.1)
Representation and Fairness	16 (6.1)	8 (7.1)
Accountability	6 (2.3)	3 (2.7)
Ethical Decision Making	3 (1.1)	0 (0.0)
**Development and Deployment**	53 (20.3)	21 (18.6)
Clinical Validation	20 (7.6)	3 (2.7)
Automation and Technological Impact	10 (3.8)	4 (3.5)
Integration and Interoperability	3 (1.1)	0 (0.0)
Socioeconomic and Administrative	5 (1.9)	1 (0.9)
Design and Development	15 (5.7)	13 (11.5)
**Certification**	22 (8.4)	10 (8.8)
Organization	15 (5.7)	10 (8.8)
Oversight and Monitoring	7 (2.7)	0 (0.0)
**Total**	**261**	**113**

## Data Availability

No new data were created or analyzed in this study. Data sharing is not applicable to this article.

## Supplementary Materials

The following supporting information can be downloaded at: https://www.mdpi.com/article/10.3390/healthcare14172865/s1, File S1: Individual Challenges and Recommendations.

## Abbreviations

The following abbreviations are used in this manuscript:

AI	Artificial Intelligence
ATMP	Advanced Therapy Medicinal Product
DiGA	Digitale Gesundheitsanwendungen (Digital Health Applications)
EFS	Early Feasibility Study
EHDS	European Health Data Space
EMA	European Medicines Agency
EU	European Union
Eudamed	European Database on Medical Devices
FDA	Food and Drug Administration (USA)
GDPR	General Data Protection Regulation
HIPAA	Health Insurance Portability and Accountability Act (USA)
IEC	International Electrotechnical Commission
ISO	International Organization for Standardization
IVD	In Vitro Diagnostic
IVDR	In Vitro Diagnostic Medical Devices Regulation (EU) 2017/746
LIME	Locally Interpretable Model-Agnostic Explanations
LLM	Large Language Model
MD	Medical Device
MDCG	Medical Device Coordination Group
MDR	Medical Devices Regulation (EU) 2017/745
MDSW	Medical Device Software
MedMLOps	Medical Machine-Learning Operations
OECD	Organisation for Economic Co-operation and Development
PCCP	Predetermined Change Control Plan
PICOC	Population, Intervention, Comparison, Outcomes, and Context
PMS	Post-Market Surveillance
PRISMA-ScR	Preferred Reporting Items for Systematic reviews and Meta-Analyses extension for Scoping Reviews
RQ	Research Question
SaMD	Software as a Medical Device
SME	Small and Medium-sized Enterprise
TRIPOD	Transparent Reporting of a multivariable prediction model for Individual Prognosis Or Diagnosis
WHO	World Health Organization
